# The Weak Visible n → π* Transition of CF_3_NO: Vertical Accuracy versus Spectral Agreement

**DOI:** 10.1021/acsomega.6c11480

**Published:** 2026-10-09

**Authors:** Gabrielly da S. Hage, Pedro Henrique R. de Oliveira, Glaucio B. Ferreira, Henrique C. Silva Junior

**Affiliations:** † Departamento de Química Fundamental, 67825 Universidade Federal Rural do Rio de Janeiro (UFRRJ), BR-465, Km 07, Seropédica Rio de Janeiro 23890-000, Brazil; ‡ Instituto de Química, Universidade Federal Fluminense, Outeiro de S. João Batista s/n, Centro, Niterói, Rio de Janeiro 24220-141, Brazil

## Abstract

Trifluoronitrosomethane (CF_3_NO), one of the few relatively stable blue gases, owes its color to a weak, structured n → π* absorption near 690 nm. Its small size suggests a straightforward benchmark, yet vertical excited-state methods place this transition very differently. We investigate this discrepancy by treating the vertical excitation, the 0–0 transition, and the finite-temperature band envelope as distinct observables. The benchmark combines full linear-response TD-DFT and TDA for five functionals with EOM-CCSD and EOM-CC3 vertical references, together with CCSD­(T), CASSCF/NEVPT2, spin–orbit, structural, and vibronic analyses. At the reference geometry, S_0_ is predominantly closed-shell, whereas S_1_ is an isolated, spin-pure state dominated by n­(N) → π*­(N=O); these diagnostics argue against pronounced multireference character, ambiguous state identity, or appreciable spin–orbit mixing as dominant explanations of the method dependence at the reference structure. EOM-CC3/aug-cc-pVTZ gives 1.974 eV, and EOM-CCSD lies only about 0.03 eV higher. Full LR-TDDFT is 0.142–0.231 eV lower (red-shifted), whereas TDA raises the same state by 0.102–0.179 eV and brings every tested functional closer to the coupled-cluster reference. The jet-cooled 0–0 origin (1.727 eV) and strongest room-temperature feature (1.789 eV) lie well below the vertical region. The local harmonic vibronic models also underestimate the 0–0 energy and recover the gross experimental envelope only after empirical alignment and broadening. CF_3_NO therefore provides a compact benchmark in which vertical accuracy, state character, and spectral agreement can be separated quantitatively rather than collapsed into a single comparison.

## Graphical abstract

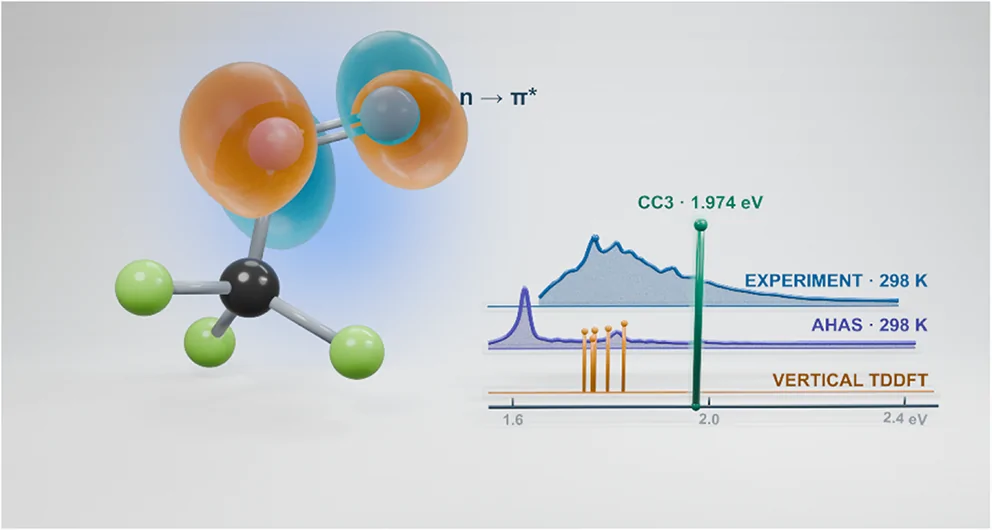

## Introduction

Trifluoronitrosomethane (CF_3_NO), one of the few relatively stable blue gases, owes its striking color to a weak absorption at the red edge of the visible spectrum. The gas-phase absorption forms a structured band with prominent maxima near 693 and 673 nm and vibrational progressions involving the N=O chromophore.^1,2^ The band has long been assigned to the spin- and symmetry-allowed S_1_(^1^A″) ← S_0_(^1^A′) n → π* excitation localized on the nitroso group.^1,3,4^ Its low intensity is consistent with limited overlap between the participating orbitals, whereas the vibrational structure points to substantial nuclear reorganization upon excitation.^1,2^

That mixture of electronic and vibrational structure matters when calculations are compared with experiment. A vertical excitation energy is defined at the S_0_ geometry and provides a clean basis for comparing electronic-structure methods, but it is not a simulated experimental absorption spectrum. The 0–0 energy includes structural relaxation and zero-point contributions, while the position of a finite-temperature band also reflects its vibronic envelope and broadening.^5–7^ Earlier TD-DFT studies of nitroso compounds, including CF_3_NO itself, used the strongest experimental feature as a practical reference for vertical energies.^8,9^ For a structured band such as that of CF_3_NO, however, agreement with λ_max_ alone does not establish that the vertical energy is accurate.

The electronic character of CF_3_NO remains unsettled in the literature. Tse and Goddard concluded that a proper description of the ground and lowest excited states requires at least a two-configuration wave function and that multireference CI is needed for the transition energy.^10^ By contrast, Azarias et al. identified predominantly single-reference character in their active-space analysis and obtained a narrow 1.938–1.973 eV range across CC2, CCSD, CCSDR(3), and CC3.^11^ The contradiction is striking: CF_3_NO is small and its lowest excitation has a chemically intuitive n → π* assignment, while wave function and density-functional treatments disagree over its underlying character. It therefore provides a direct test of whether the reported difficulty arises from genuine multireference character or from the limitations of single-reference descriptions of a demanding but well-defined excited state.

Distinguishing vertical excitation energies from experimental spectral features is an established requirement in benchmarking, including the treatment of relaxation and vibrational effects by Goerigk et al.^12^ In this work we use CF_3_NO as a compact test of vertical excited-state methods while treating the relevant electronic and spectroscopic quantities separately. Full linear-response TD-DFT and TDA are evaluated across a controlled basis-set sequence and benchmarked against canonical EOM-CCSD, EOM-CC3, and published high-level vertical references rather than the experimental maximum alone. Canonical CCSD­(T) diagnostics and CASSCF/NEVPT2/SOC calculations establish the electronic character and isolation of the target state, while method-consistent structural and vibronic calculations examine how nuclear relaxation connects the vertical transition with the observed band. For CF_3_NO, this combination directly tests the reported need for a multireference description and separates errors in the vertical excitation from the effects of structural relaxation and vibronic structure on the observed band.

## Computational Methods

Electronic-structure calculations were performed with ORCA 6.1.0 and 6.1.1.^13,14^ The closed-shell S_0_ geometry of CF_3_NO was optimized in the gas phase at the revDSD-PBEP86-D4/def2-QZVP level.^15^ This structure was used for the common-geometry vertical benchmark; method-specific S_0_ and S_1_ structures were generated only for the geometry-sensitivity and vibronic analyses. During optimization no symmetry constraint was imposed. Cartesian coordinates and calculation-specific settings are provided in the Supporting Information.

The electronic and spectroscopic comparisons were defined separately. Vertical excitation energies are S_1_–S_0_ differences at the S_0_ reference geometry. Published 0–0 assignments, the jet-cooled origin, and the room-temperature gas-phase band maxima were treated as distinct spectroscopic observables.^1,3,4,16,17^ The experimental absorption profile was digitized from the spectrum reported by Mason^2^ and considered together with the established gas-phase and jet-cooled assignments. Calculated vibronic envelopes were compared with the experimental band shape rather than used as substitutes for the vertical benchmark.

The room-temperature experimental profile was generated from the digitized Mason band maxima using shape-preserving interpolation.^2,17^ For Figure 3, the experimental and calculated profiles were normalized and displayed with a common Gaussian full width at half-maximum of 80 cm^–1^. Empirically aligned and broadened comparisons were used only as a Supporting Information shape diagnostic and did not enter any energy ranking.

Full linear-response TD-DFT and TDA calculations were carried out with B3LYP, CAM-B3LYP, R2SCAN-QIDH, ωB97M-D4, and ωB97X-D4.^18–28^ Calculations were performed with def2-TZVP, def2-TZVPP, def2-QZVP, def2-QZVPPD, and aug-cc-pVQZ.^29–31^ Because def2-TZVP and def2-TZVPP generate the same orbital basis for C, N, O, and F in ORCA, the convergence analysis treats them as one of four distinct orbital-basis representations. Forty singlet roots were obtained for each TDDFT/TDA calculation.

The D4 term does not contribute directly to a fixed-geometry linear-response excitation energy; the suffix is retained as part of the functional name, and dispersion enters only through method-specific optimized geometries where applicable. R2SCAN-QIDH and ωB97M-D4 depend explicitly on the kinetic-energy density τ and require the corresponding current-density contribution in the time-dependent response to preserve gauge invariance. This gauge-restoration term is not included in the ORCA implementation used here; the R2SCAN-QIDH and ωB97M-D4 excitation energies are therefore reported for completeness but are not used alone to establish an absolute accuracy ranking.^32,33^ The B3LYP, CAM-B3LYP, and ωB97X-D4 functionals used for the vibronic calculations do not depend on τ and are therefore unaffected by this specific current-density omission.

The target state was followed through its dominant n­(N) → π*­(N=O) natural-transition-orbital pair, energy ordering, and oscillator strength. Excitation energies were the primary benchmark quantities. Length- and velocity-representation oscillator strengths, when available, were used only to verify state identity and transition-moment consistency and are reported in the Supporting Information.^34,35^

Canonical EOM-CCSD calculations were performed at the common reference geometry with the same four distinct orbital-basis representations, targeting ten singlet roots and using converged right and left eigenvectors with exact transition-density matrices.^36^ The EOM-CC3/aug-cc-pVTZ calculation was performed with eT software v2.5.1 at the final postcycle geometry from the same unsymmetrized revDSD optimization, targeting three singlet roots with the implementation of Paul et al.^37,38^ The right and left excited-state vectors, biorthonormalization, and EOM transition moments converged for all three roots; the state-resolved left-vector compositions and length-, velocity-, and mixed-gauge oscillator strengths are reported in the Supporting Information. The EOM-CCSD calculations froze 12 core electrons, whereas EOM-CC3 correlated all 48 electrons and used an integral Cholesky threshold of 10^–4^. The CCSD–CC3 energy separation therefore includes differences in basis, core treatment, and evaluated geometry and is not an isolated triples increment.

Geometry sensitivity was isolated with CAM-B3LYP/def2-TZVP by evaluating full TDDFT and TDA at both the common revDSD-PBEP86-D4 structure and a CAM-B3LYP-optimized S_0_ structure.

State-averaged CASSCF and NEVPT2 were used to test the character and isolation of the low-lying states.^39–43^ The primary diagnostic employed CAS­(8,12)/aug-cc-pVQZ, with equal averaging over 10 singlet and 10 triplet roots, followed by partially contracted NEVPT2; the active orbitals span the nitroso lone-pair, π/π*, and associated valence space. Active-space sensitivity was examined in the Supporting Information with CAS­(8,7) and CAS­(8,8)/def2-TZVP calculations prepared from the same SA-CASSCF­(10,10) natural-orbital protocol and followed by strongly contracted NEVPT2. Spin–orbit coupling was evaluated by quasi-degenerate perturbation theory for each active-space model.

Ground-state single-reference character was assessed from the T_1_ diagnostic and the corresponding normalized Λ_1_ singles norm of the canonical CCSD­(T)/cc-pVQZ calculation.^44,45^ A separate canonical RHF-CCSD­(T) calculation provided the S diagnostic.^46^ The converged Λ amplitudes yielded the unrelaxed CCSD­(T) density, which was analyzed together with the SCF density. NBO 7.0 analyses provided natural populations, bond indices, orbital occupancies, donor–acceptor interactions, and dipole moments.^47^

An occupation-constrained open-shell singlet ΔSCF calculation at revDSD-PBEP86-D4/def2-QZVP was used to inspect the density rearrangement associated with the n­(N) → π*­(N=O) configuration. The determinant was generated by swapping the α-HOMO and α-LUMO occupations and maintained through a maximum-overlap procedure.^48^ Charges and Mayer bond orders were evaluated from the unrelaxed MP2 electron density; atomic spin populations were evaluated from the corresponding unrelaxed MP2 spin density. This construction served only as a density-analysis model and was not used as an oscillator-strength or vertical-energy reference.

The vibronic analysis was performed with ORCA_ESD at full TDDFT/def2-TZVP using B3LYP, CAM-B3LYP, and ωB97X-D4. For each functional, the S_0_ minimum and harmonic force field were computed and the target n → π* S_1_ root was followed consistently to obtain the excited-state gradients and one-step Hessians required by the local spectral models.

Franck–Condon and first-order Herzberg–Teller absorption profiles were generated within the harmonic path-integral framework at 0 and 298.15 K.^5,6^ In the vertical-gradient (VG) model, the S_1_ gradient displaces the excited-state surface while the S_0_ Hessian is retained. In the adiabatic-Hessian-after-a-step (AHAS) model, one controlled step is taken toward the S_1_ minimum and the excited-state Hessian is recalculated there. ORCA_ESD provided a model-consistent harmonic 0–0 energy for each description.

A fully optimized adiabatic-Hessian construction was also attempted, but the large S_0_/S_1_ RMSD displacement made the linear Duschinsky mapping between the independently optimized minima unreliable for CF_3_NO. It was therefore excluded from spectral interpretation; only the raw calculations and failure diagnostics are retained in the Supporting Information. Duschinsky rotation was included in AHAS, where both Hessians are defined. An exploratory CC3-anchored 0–0 correction based only on stationary-point energies and zero-point contributions is reported in the Supporting Information; its sensitivity to the geometry convention prevents its use as the primary spectral benchmark.

The VG and AHAS profiles were examined at their native fine resolution and after the common 80 cm^–1^ Gaussian display convolution. A separate optimization of rigid shift and broadening was performed only to test gross envelope similarity; those fitted parameters are reported in the Supporting Information and are not treated as predictions.

Excitation energies and signed differences were analyzed primarily in eV and reported also in cm^–1^; wavelengths were retained only as spectroscopic conversions. No wavelength-domain averages or rankings were used. Basis dependence was described by energy spans across the four distinct orbital representations. Structural parameters are reported in Å and degrees, dipole moments in Debye, atomic charges in units of the elementary charge, and oscillator strengths and bond orders as dimensionless quantities. Unrounded values and calculation-specific settings are provided in the Supporting Information.

## Results and Discussion

### Electronic Structure and State Character of CF_3_NO

Before benchmarking the vertical excitation energy, the electronic character of the states involved must first be established. This point remains disputed for CF_3_NO: Tse and Goddard concluded that both S_0_ and the lowest excited state require at least a two-configuration treatment, whereas Azarias et al. later found predominantly single-reference character.^10,11^ The analysis below therefore tests whether the visible transition is intrinsically multireference or instead a well-defined n­(N) → π*­(N=O) excitation whose energy is unusually method-sensitive.

The earlier multireference assignment was tied in part to the unusually long C–N bond, making the S_0_ structure the natural starting point. At revDSD-PBEP86-D4/def2-QZVP, r­(C–N) = 1.526 Å, r­(N=O) = 1.194 Å, and ∠C–N=O = 112.2°. Gas-phase spectroscopy gives the same bent nitroso framework: the experimental angle is 112.4°, and the C–F, N–O, and C–N distances differ from the calculated values by no more than 0.014 Å.^49^ The geometry used for the vertical analysis, then, closely reproduces the observed molecular structure.

At this geometry, the ground-state density is strongly polarized across the CF_3_–N=O framework. NPA assigns +1.071 e to C, +0.100 e to N, and −0.206 e to O. The three fluorine atoms leave the carbon atom markedly electron deficient, while the opposite signs on N and O retain the internal polarity of the nitroso unit. The n­(N) donor and π*­(N=O) acceptor are therefore embedded in the polarization of the complete CF_3_–N=O framework, not in an electronically isolated NO fragment.

That local polarization is largely hidden in the molecular dipole. The calculated value is only 0.268 D, close to the experimental gas-phase value of 0.18 D,^50^ because the bond and lone-pair contributions largely cancel as vectors (Table 1). Thus, the small total dipole does not imply a weakly polarized chromophore; it merely conceals the local charge separation from which the visible excitation begins.

**Table 1:** Selected S_0_ Structural and Electronic Descriptors of CF_3_NO at the revDSD-PBEP86-D4/def2-QZVP Level^a^

Quantity	S_0_ value
r(C–N)/Å	1.526
r(N=O)/Å	1.194
r(C–F) range/Å	1.316–1.324
∠C–N=O/°	112.2
qNPA(C)/e	+1.071
qNPA(N)/e	+0.100
qNPA(O)/e	–0.206
qNPA(CF_3_ fragment)/e	+0.106
qNPA(NO fragment)/e	–0.106
μ/D	0.268

a NPA charges were obtained from the unrelaxed MP2 electron density associated with the revDSD-PBEP86-D4 double-hybrid calculation.

The occupation-constrained ΔSCF density shows how this polarized framework responds to the n­(N) → π*­(N=O) excitation at the fixed S_0_ geometry. Relative to S_0_, nitrogen accumulates 0.161 e of electron density, oxygen loses 0.101 e, and the CF_3_ fragment loses only 0.060 e (Figure 1 and Table 2). The increase at nitrogen is consistent with the substantial N contribution to the π*­(N=O) acceptor; the self-consistent difference density also includes the accompanying orbital relaxation. The bonding response is correspondingly localized: the Mayer N=O bond order decreases from 1.751 to 1.507, whereas C–N changes only from 0.935 to 0.911. The immediate vertical response is therefore dominated by N=O antibonding and N/O open-shell polarization, with only modest redistribution through CF_3_. These descriptors locate the excitation on the nitroso group, but they do not determine whether S_0_ and S_1_ require a multireference wave function.

**Figure 1:**
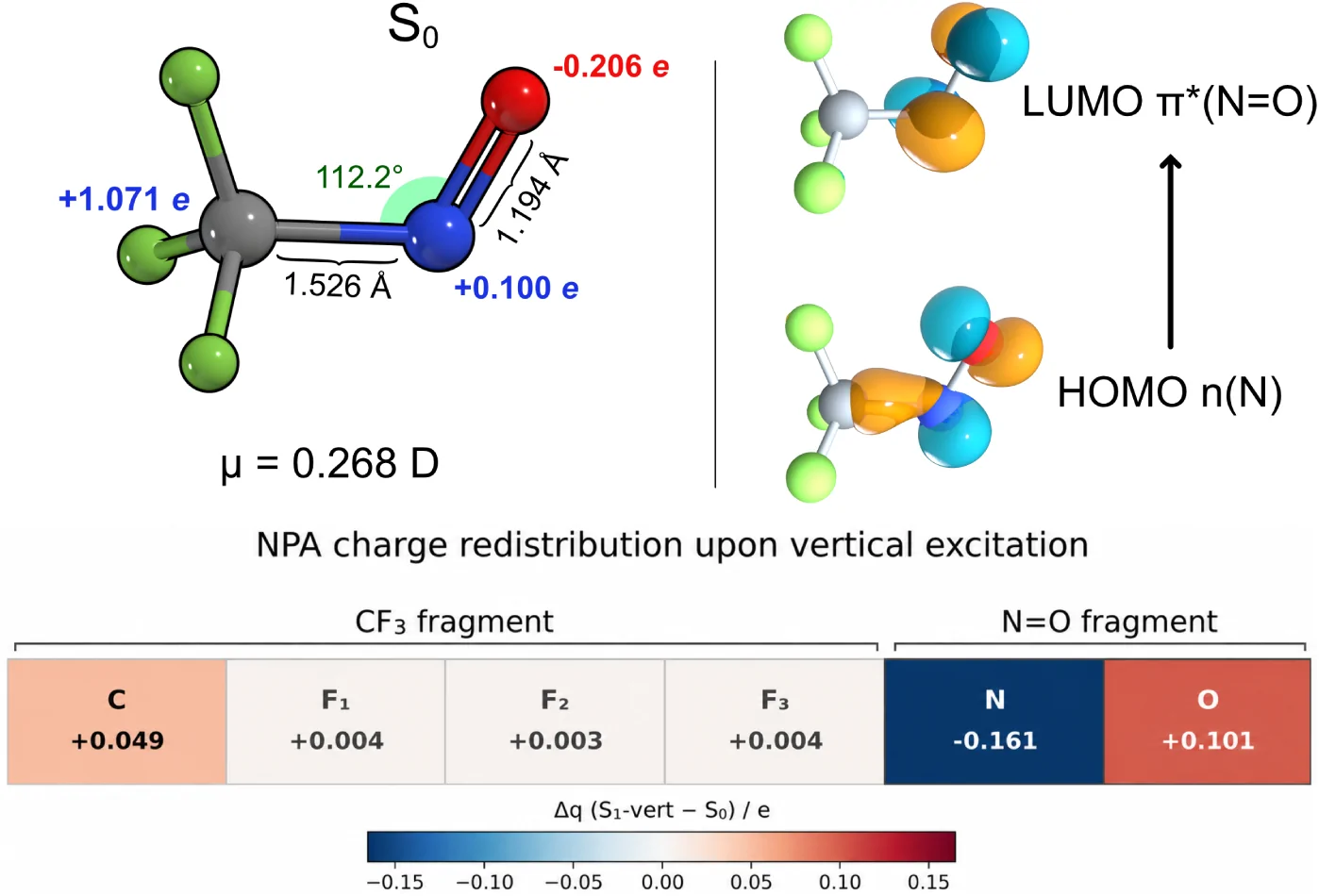
Structural and electronic descriptors of CF_3_NO. Left: optimized S_0_ structure at the revDSD-PBEP86-D4/def2-QZVP level with selected NPA charges, bond lengths, angle, and dipole moment. Right: HOMO n­(N) and LUMO π*­(N=O) orbitals defining the target n­(N) → π*­(N=O) excitation. Bottom: vertical ΔSCF NPA charge differences, Δq = q­(S_1_-vert) – q­(S_0_); blue and red denote electron-density gain and loss, respectively.

**Table 2:** Vertical ΔSCF Density Descriptors for the Imposed n­(N) → π*­(N=O) Configuration of CF_3_NO at the S_0_ Geometry^a^

Descriptor	S_0_	S_1_-vert	Change/interpretation
qNPA(N)/e	+0.100	–0.061	N gains electron density
qNPA(O)/e	–0.206	–0.105	O becomes less negative
spin density (N)	0.000	+0.589	localized α excess on N
spin density (O)	0.000	–0.429	localized β excess on O
Mayer BO (C–N)	0.935	0.911	minor vertical weakening
Mayer BO (N=O)	1.751	1.507	strong π* antibonding response

a Charges and Mayer bond orders were obtained from the unrelaxed MP2 electron density, while atomic spin populations were obtained from the corresponding unrelaxed MP2 spin density associated with the vertical revDSD-PBEP86-D4 ΔSCF calculation.

Having established where the imposed excitation redistributes the density, we next ask whether the equilibrium ground state lies beyond a reliable single-reference description. Canonical RHF-CCSD­(T)/cc-pVQZ gives the T_1_ diagnostic = 0.01344 and a normalized Λ_1_ singles norm of 0.01069, while the complementary S diagnostic is 0.690097.^46^ Taken together, these values provide no indication that the RHF determinant is qualitatively inadequate or that the single-reference coupled-cluster solution approaches a breakdown regime. Thus, the equilibrium S_0_ state shows no evidence of pronounced multireference character. Whether its wave function nevertheless contains substantial mixing with a specific secondary valence configuration is a separate question, addressed directly by the active-space expansion.

The active-space expansion supplies the direct configurational test left open by the coupled-cluster diagnostics. In CAS­(8,12), the closed-shell configuration carries a weight of 0.897 in S_0_. The largest secondary configuration contributes only 0.048, and the remaining weight is divided among even smaller terms. The 0.103 outside the leading configuration therefore reflects distributed valence correlation rather than a second reference of comparable importance. S_0_ is dynamically correlated, but its wave function remains organized around a single closed-shell configuration.

The literature disagreement, however, concerns both states. An adequate S_0_ reference would not exclude the possibility that occupation of π*­(N=O) produces a strongly mixed S_1_. The active-space result shows that the configurational hierarchy is retained: the n­(N) → π*­(N=O) configuration carries a weight of 0.883, while the largest secondary contribution is only 0.045. EOM-CC3/aug-cc-pVTZ reaches the same conclusion from a different wave function representation, yielding right- and left-hand S_1_ energies of 1.973913 and 1.973858 eV and singles characters of 96.5% and 93.0%, respectively. The CASSCF weight and the EOM-CC3 singles fractions are not directly equivalent quantities, but both show that correlation modifies S_1_ without displacing the n­(N) → π*­(N=O) excitation as its dominant character.

A dominant configuration alone does not establish that the assigned root is isolated. A recognizable n → π* state could still be perturbed by nearby singlets or mixed appreciably with a low-lying triplet. The NEVPT2 and spin–orbit results rule out both possibilities. At the CAS­(8,12)/aug-cc-pVQZ PC-NEVPT2 level, S_1_ lies at 2.009 eV and S_2_ at 4.843 eV, leaving a separation of 2.834 eV. T_1_ lies at 1.111 eV, giving an S_1_–T_1_ separation of 0.899 eV, while the QDPT eigenstate correlated with S_1_ retains 99.9997% singlet character. The visible state is thus well separated within the singlet manifold and essentially unmixed with T_1_.

The apparent contradiction between Tse/Goddard and Azarias et al. is resolved once structural sensitivity is separated from reference character. Tse and Goddard correctly identified the structural importance of C–N-antibonding mixing and concluded that S_0_ and the lowest excited state required at least a two-configuration self-consistent-field (TCSCF) description, with multireference CI needed for the transition energy.^10^ Yet the secondary configuration in their own two-configuration wave function carried only 1.8% of the total weight. Their larger CAS­(8,9) expansions were likewise dominated by a single configuration, with leading coefficients of 0.954 for S_0_ and 0.965 for S_1_, corresponding to weights of 0.910 and 0.931.^10^ Their calculations therefore already contain the crucial distinction: a small antibonding contribution can exert a disproportionate influence on the C–N bond without becoming a codominant electronic reference. The present coupled-cluster diagnostics, CASSCF weights, EOM-CC3 singles character, NEVPT2 state separations, and spin–orbit purity establish the state character directly. They resolve that question in favor of the later assessment of Azarias et al.^11^ while preserving Tse and Goddard’s structural insight. At the reference geometry, CF_3_NO is configuration-sensitive but not strongly multireference: S_0_ remains predominantly closed shell, and S_1_ is a well-defined, isolated n­(N) → π*­(N=O) singlet. The vertical benchmark that follows therefore tests how accurately different methods place the energy of this state, not whether the state itself is ill-defined.

### Vertical Benchmark of the Visible n → π* Transition

As the state-character analysis removes the central ambiguity from the benchmark the methods can now be compared on where they place the same isolated n­(N) → π*­(N=O) state. Our EOM-CC3/aug-cc-pVTZ calculation gives 1.974 eV, essentially at the upper edge of the 1.938–1.973 eV coupled-cluster interval reported by Azarias et al.^11^ Canonical EOM-CCSD gives 2.005–2.006 eV across the four distinct orbital-basis representations, only 0.031–0.032 eV higher. The present EOM-CC3 and EOM-CCSD calculations therefore place the vertical transition near 1.97–2.01 eV.

Energy variation across the tested TDDFT/TDA and EOM-CCSD orbital-basis representations is small. Across the four distinct orbital-basis representations, every TDDFT/TDA series spans less than 10 meV, whereas EOM-CCSD varies by only 1.2 meV. def2-TZVP and def2-TZVPP are counted as a single basis point because they generate the same orbital basis for C, N, O, and F in ORCA. The LR–TDA energy shifts shown in Figure 2 and Table 3 are substantially larger than this observed basis variation.

**Figure 2:**
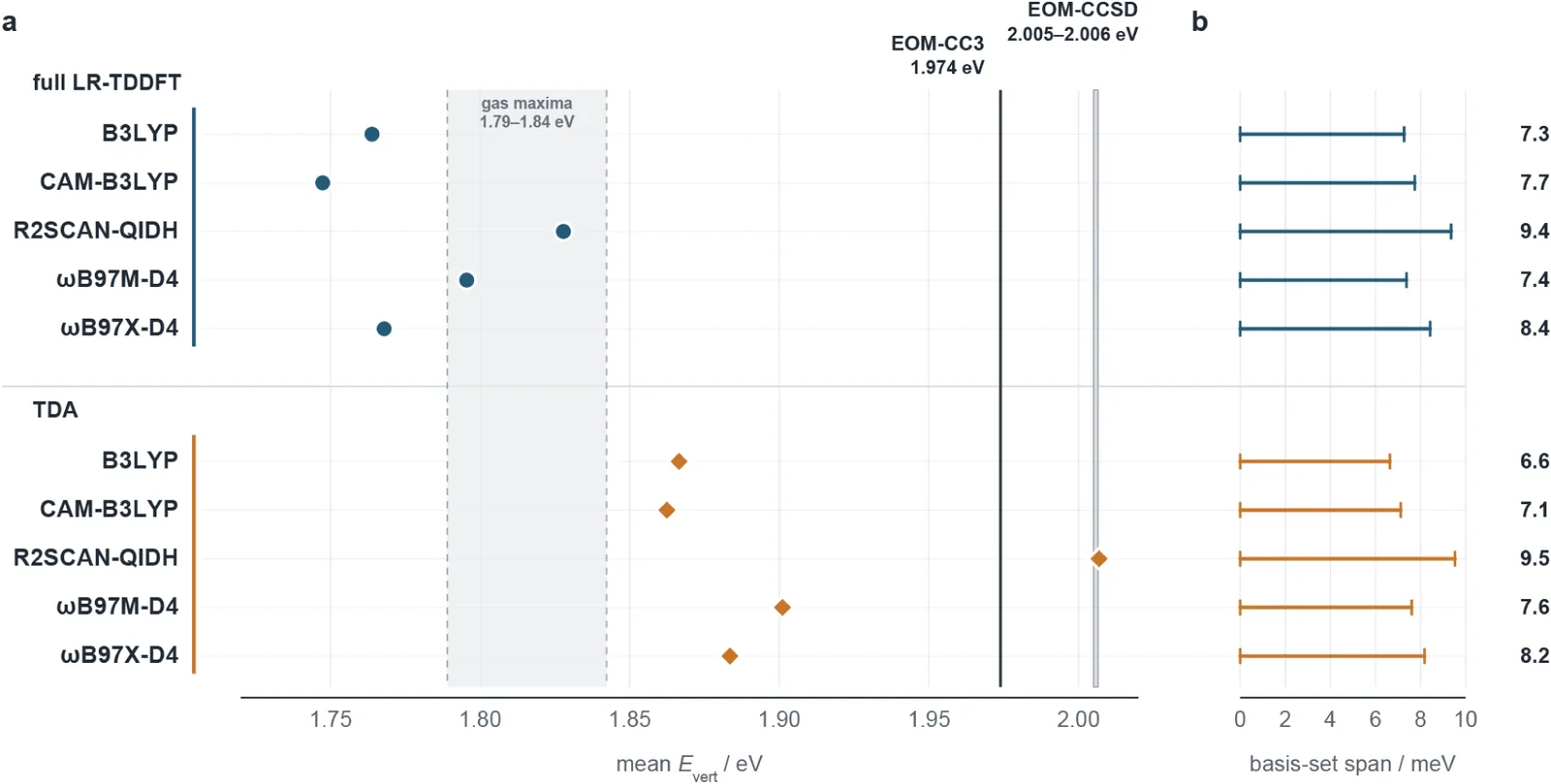
Vertical S_1_ n­(N) → π*­(N=O) excitation energies of CF_3_NO. (a) Mean full LR-TDDFT (blue circles) and TDA (orange diamonds) energies over the four distinct orbital-basis representations. The pale gray band spans the gas-phase maxima at 1.79–1.84 eV (693–673 nm); the dark line and narrow gray bar indicate EOM-CC3/aug-cc-pVTZ (1.974 eV) and the EOM-CCSD range (2.005–2.006 eV), respectively. (b) Corresponding basis-set spans.

**Table 3:** Vertical S_1_ n­(N) → π*­(N=O) Excitation Energies of CF_3_NO^a^

Method	Formalism	*E*_vert_ range (eV)	Basis span (meV)	Δ*E* vs CC3 (eV)	Δ*E*_TDA–LR_ (eV)
**B3LYP**	full LR-TDDFT	1.760–1.767	7.3	–0.214 to −0.207	—
	TDA	1.863–1.869	6.6	–0.111 to −0.105	+0.102 to +0.103
**CAM-B3LYP**	full LR-TDDFT	1.743–1.751	7.7	–0.231 to −0.223	—
	TDA	1.858–1.865	7.1	–0.116 to −0.109	+0.115
**R2SCAN-QIDH**	full LR-TDDFT	1.822–1.831	9.4	–0.152 to −0.142	—
	TDA	2.001–2.011	9.5	+0.027 to +0.037	+0.179
**ωB97M-D4**	full LR-TDDFT	1.791–1.798	7.4	–0.183 to −0.176	—
	TDA	1.896–1.904	7.6	–0.078 to −0.070	+0.105 to +0.106
**ωB97X-D4**	full LR-TDDFT	1.763–1.771	8.4	–0.211 to −0.203	—
	TDA	1.879–1.887	8.2	–0.095 to −0.087	+0.115 to +0.116
EOM-CCSD	EOM	2.005–2.006	1.2	+0.031 to +0.032	—
**EOM-CC3**	**EOM**	**1.974**	—	**reference**	—

a Ranges and basis spans refer to the four distinct orbital-basis representations. ΔE versus CC3 is signed relative to the EOM-CC3 value of 1.974 eV, and ΔETDA–LR compares paired TDA and full LR-TDDFT results. Complete basis-resolved data and oscillator strengths are given in the Supporting Information.

Relative to CC3, full LR-TDDFT is systematically lower in energy (red-shifted). Across the five functionals, the values span 1.743–1.831 eV, corresponding to red shifts of 0.142–0.231 eV; none reaches the high-level vertical region. R2SCAN-QIDH and ωB97M-D4 give the highest full-response energies, although the uncorrected τ-dependent response prevents their ordering from defining an absolute accuracy ranking. The shaded gas-phase interval in Figure 2 (1.79–1.84 eV) contains the four-basis means of both R2SCAN-QIDH and ωB97M-D4.^1,2^ Their basis-resolved vertical energies nevertheless remain 0.142–0.183 eV below CC3. This overlap is therefore deceptive rather than evidence of vertical accuracy: the electronic-energy error compensates for the physical displacement between the vertical transition and the vibronic band.

TDA changes this pattern systematically. Removing excitation-de-excitation coupling raises the matched state by 0.102–0.179 eV, more than ten times the largest basis effect. This upward tendency is consistent with established behavior of CIS/TDA-type response for weak n → π* transitions and is treated here as a quantitative confirmation for CF_3_NO rather than as a new generic failure mode.^11,19^ The TDA values consequently move closer to CC3 for every functional: R2SCAN-QIDH is 0.027–0.037 eV higher (blue-shifted), whereas the remaining four are 0.070–0.116 eV lower (red-shifted). This improvement is specific to the present transition and should not be read as a general superiority of TDA. Here, the upward TDA shift happens to compensate for the low-energy bias of the full-response results.

Throughout the comparison, the target root retains the same n­(N) → π*­(N=O) NTO character. The EOM-CC3 transition moments give oscillator strengths of 2.53 × 10^–4^ (length gauge) and 2.47 × 10^–4^ (velocity gauge), consistent with the same weakly allowed character. The energy shifts therefore reflect how each formalism places the state, not root switching or a change in its identity. Complete oscillator-strength data are reported in the Supporting Information and are not used to rank the excitation energies.^34,35^

Taken together, the vertical results demonstrate substantial sensitivity to the response approximation for the same identified electronic state. Energy changes across the tested orbital bases are small, EOM-CCSD and EOM-CC3 anchor the vertical transition near 1.97–2.01 eV, and full LR-TDDFT remains red-shifted from that region even when it appears close to the experimental band. TDA moves the same state upward and closer to the vertical reference. This paired comparison is the central result of the benchmark: agreement with the visible band can coexist with a substantial underestimate of the vertical excitation energy because spectral agreement and vertical accuracy are not interchangeable. CF_3_NO thus provides a controlled molecular case in which response-method sensitivity can be assessed alongside basis-set and state-character diagnostics. These comparisons constrain the explanation of the method dependence without identifying a unique microscopic error in each functional. The remaining separation between the high-level vertical energy and the observed red-edge band is not a problem of state identity; it must be sought in structural relaxation, zero-point contributions, and vibronic intensity redistribution, examined next.

### From the Vertical Excitation to the Vibronic Band

Having established the identity and vertical energy of S_1_, the remaining question is how this electronic transition produces the observed spectrum. EOM-CC3 places the vertical excitation at 1.974 eV, whereas the jet-cooled 0–0 transition occurs at 1.727 eV and the strongest room-temperature feature at 1.789 eV, 500 cm^–1^ above the jet-cooled 0–0 origin.^2,16,17^ The room-temperature maximum is therefore distinct from both the vertical excitation and the 0–0 transition. A meaningful spectral test must reproduce the structural relaxation and redistribution of intensity that separate these observables.

To describe the vibronic band without relying on a harmonic mapping between the widely separated S_0_ and S_1_ minima, we used two local harmonic models. In the vertical-gradient (VG) model, the S_1_ gradient displaces the excited-state surface while the S_0_ Hessian is retained. In the adiabatic-Hessian-after-a-step (AHAS) model, one step is taken toward the S_1_ minimum and the excited-state Hessian is recalculated at the displaced geometry, allowing the curvature and normal-mode mixing to respond.^5,6^ Both models include Franck–Condon (FC) and first-order Herzberg–Teller (HT) contributions at 0 and 298 K. The fully optimized adiabatic-Hessian treatment is not included in the main comparison because the large S_0_/S_1_ displacement makes the linear Duschinsky mapping unreliable; the corresponding diagnostics are given in the Supporting Information.

Figure 3a shows that the proximity of several full-response TDDFT vertical energies to the visible band maximum does not translate into predictive agreement with the spectrum. On the same functional surfaces, the VG and AHAS models give harmonic 0–0 energies of 1.61–1.66 eV, which are 0.07–0.12 eV lower (red-shifted) than the experimental jet-cooled 0–0 energy of 1.727 eV. The calculated 298 K envelopes are therefore also displaced to lower energy. The apparent agreement at the level of a single vertical energy disappears when each calculated quantity is compared with the corresponding experimental observable.

**Figure 3:**
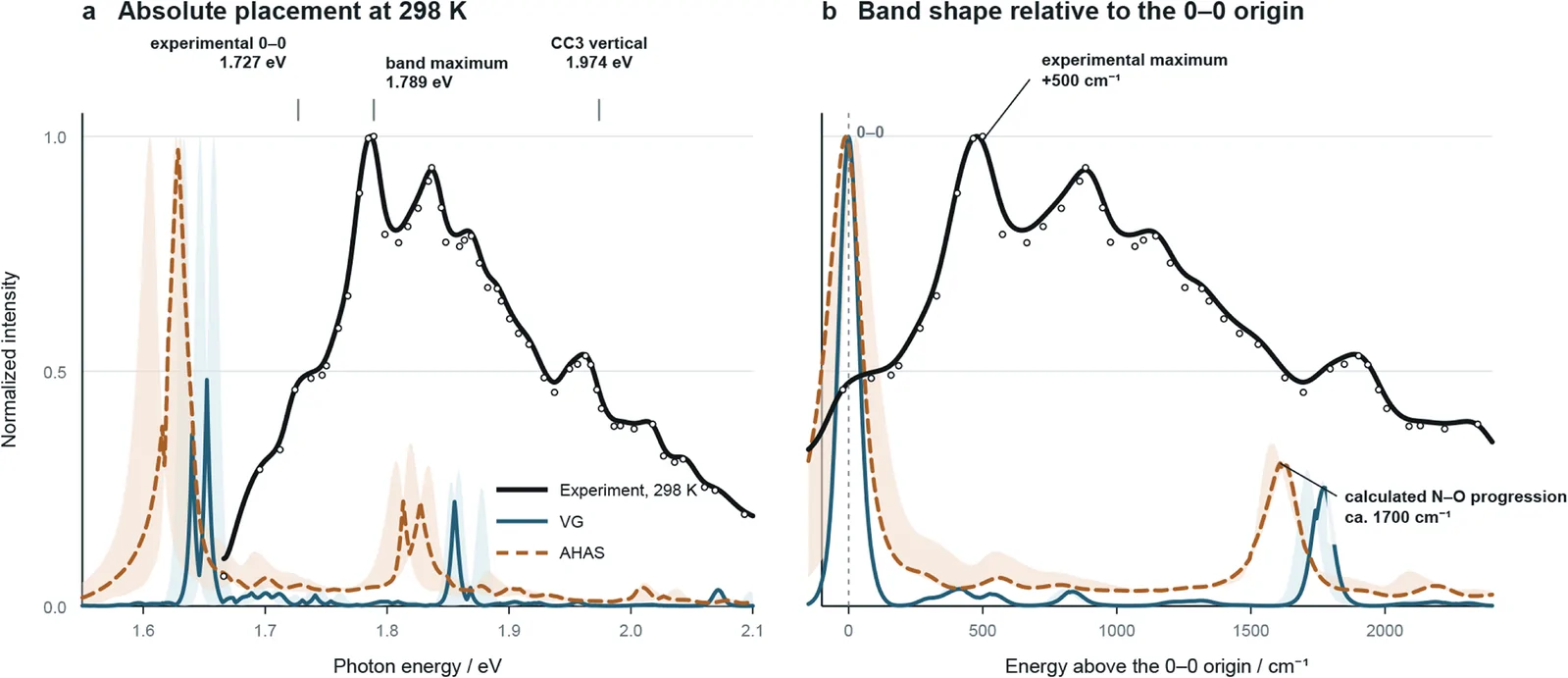
Experimental and calculated vibronic profiles for the CF_3_NO n­(N) → π*­(N=O) absorption. (a) Absolute 298 K spectra, with the EOM-CC3 vertical energy, experimental 0–0 origin, and room-temperature band maximum marked separately. The experimental points are the digitized band maxima reported by Mason (ref^2^). For both VG and AHAS, lines show the median and shaded regions span the B3LYP, CAM-B3LYP, and ωB97X-D4 profiles. (b) The same profiles referenced to their respective 0–0 energies. All profiles are displayed with an 80 cm^–1^ Gaussian FWHM. Experimental data adapted from ref^2^ with permission from the Royal Society of Chemistry.

Referencing each calculated spectrum to its own 0–0 energy removes this absolute offset and isolates the band shape (Figure 3b). The experimental envelope reaches its maximum about 500 cm^–1^ above the jet-cooled 0–0 origin. In every VG and AHAS spectrum, by contrast, the 0–0 band remains dominant and the next substantial progression appears near 1700 cm^–1^. The 0 K profiles in the Supporting Information show the same hierarchy: thermal population broadens the envelope but does not generate the missing low-frequency intensity.

The mode analysis explains the calculated pattern. Across the three functionals, roughly four-fifths of the reorganization energy is assigned to the N–O stretching coordinate at 1710–1770 cm^–1^. That response is consistent with the π*­(N=O) population and N–O weakening established above, but it drives intensity into the high-frequency progression. The experimental band develops much earlier: Mason^2^ reported recurring intervals near 450–500 and 700–800 cm^–1^, and the jet-cooled spectrum later established a prominent hindered-torsional progression.^16^ The local harmonic models therefore capture the N–O response while underrepresenting the low-frequency motion that moves intensity away from the 0–0 band. HT coupling redistributes the intensity, but it does not reverse this hierarchy.

What CF_3_NO adds is not another demonstration that vertical and 0–0 energies differ. Here, the state identity, single-reference character, and high-level vertical energy are fixed independently. That control separates two failures that a direct comparison with λ_max_ collapses into one number: full LR-TDDFT underestimates the electronic gap, while the local harmonic spectra retain too much intensity at the origin and in the N–O stretching progression. Apparent agreement with the observed color is therefore obtained by compensation, not by a uniformly accurate description of the electronic and vibrational problem. After rigid alignment and empirical broadening, the VG and AHAS profiles reproduce the gross experimental envelope closely (Supporting Information), showing that the local models retain useful shape information even though the substantial corrections required make the agreement diagnostic rather than predictive.

## Conclusions

CF_3_NO is demanding for reasons other than an ill-defined electronic state. At the reference geometry, S_0_ remains predominantly closed-shell and single-reference, while S_1_ is an isolated, spin-pure state dominated by the local n­(N) → π*­(N=O) excitation. The present evidence clarifies the relationship between structural sensitivity and reference character: C–N-antibonding configurations exert genuine structural leverage, but neither state is organized around competing references of comparable weight.

With that ambiguity removed, the vertical benchmark becomes direct. EOM-CC3 places S_1_ at 1.974 eV, and EOM-CCSD lies only about 0.03 eV higher, with small energy changes across the tested CCSD basis sets. Full LR-TDDFT is systematically red-shifted from this region, whereas TDA raises the same state by 0.10–0.18 eV and brings every tested functional closer to the coupled-cluster reference. This does not establish a general superiority of TDA. It shows that, for this weak n → π* transition, excitation-de-excitation coupling changes the vertical energy far more than basis enlargement once state assignment and reference character have been controlled.

The experimental color nevertheless cannot serve as a vertical benchmark. The high-level vertical excitation, the jet-cooled 0–0 origin, and the room-temperature band maximum are distinct observables. On the same TDDFT surfaces, the VG and AHAS models place the 0–0 transition too low and retain excessive intensity at the origin and in the high-frequency N–O progression, whereas the measured band develops through lower-frequency structure. Empirical alignment and broadening recover the gross envelope, confirming that the local harmonic models retain useful shape information, but the corrections required make that agreement diagnostic rather than predictive. The remaining mismatch points instead to low-frequency nuclear motion beyond the local harmonic models.

CF_3_NO therefore provides a compact benchmark in which state character, vertical accuracy, and vibronic band formation can be disentangled rather than collapsed into one wavelength comparison. For this transition, agreement with λ_max_ can coexist with an inaccurate vertical gap because the electronic-energy error compensates for the physical displacement between the vertical transition and the vibronic band. This case illustrates the established requirement to assess like observables, with vertical methods judged against vertical references and spectral models judged against origins and band shapes.

## Supplementary Material



## Notes

Detailed computational protocols; Cartesian coordinates; basis-resolved full LR-TDDFT, TDA, EOM-CCSD, and EOM-CC3 data; oscillator strengths and root assignments; CCSD­(T), ΔSCF, CASSCF/NEVPT2, active-space, and spin–orbit diagnostics; method-specific structures and 0–0 energies; VG and AHAS Franck–Condon/Herzberg–Teller spectra at 0 and 298 K; full adiabatic-Hessian diagnostics; the empirically aligned and broadened shape comparison; and the digitized experimental spectrum and figure-source data (PDF)
