# Supplementary material for: The Weak Visible n → π* Transition of CF3NO: Vertical Accuracy versus Spectral Agreement
Source: ACS Omega. 2026 Oct 9;11(41):64274–82. doi: 10.1021/acsomega.6c11480 (PMC13688469; doi:10.1021/acsomega.6c11480)
Supplement: Supplementary file 1 [file ao6c11480_si_001.pdf]

Supporting Information: The Weak Visible $n \to \pi^*$ Transition of CF_3NO: Vertical Accuracy versus Spectral Agreement

Gabrielly da S. Hage,[†] Pedro Henrique R. de Oliveira,[†] Glaucio B. Ferreira,[‡] and Henrique C. Silva Junior[*,†]

†*Departamento de Química Fundamental, Universidade Federal Rural do Rio de Janeiro (UFRRJ), BR-465, km 07, Seropédica, RJ 23890-000, Brazil*

‡*Instituto de Química, Universidade Federal Fluminense, Outeiro de S. João Batista s/n., Centro, 24220-141, Niterói, RJ, Brazil*

E-mail: henriquecsj@ufrrj.br

Supporting Information

This document gives the computational details and numerical data underlying the vertical-excitation, state-character, and vibronic analyses of CF_3NO. The vertical $S_1 \leftarrow S_0$ excitation, the jet-cooled 0–0 transition, and the 298 K band maximum are reported separately throughout. References cited by number correspond to the reference list of the main manuscript.

Contents

S1 Computational details and definitions **S-3**

S1.1 Vertical excitation calculations S-3

S1.2 Coupled-cluster and state-character calculations S-4

S1.3 Vibronic calculations . . . S-5
S1.4 Observable definitions . . . S-5

S2 Reference and method-specific structures **S-6**
S2.1 Common S_0 production structure . . . S-6
S2.2 Effect of the S_0 geometry on the vertical energy . . . S-7
S2.3 Local displacements used in the VG and AHAS models . . . S-7

S3 Experimental spectral data **S-8**

S4 Full LR-TDDFT and TDA vertical excitation results **S-9**

S5 Coupled-cluster results **S-13**
S5.1 EOM-CCSD . . . S-13
S5.2 EOM-CC3 . . . S-14

S6 Ground-state and ΔSCF descriptors **S-16**

S7 CASSCF/NEVPT2 and spin–orbit analysis **S-18**

S8 VG and AHAS vibronic calculations **S-19**

S9 Fully optimized adiabatic-Hessian analysis **S-20**
S9.1 Exploratory scalar CC3-anchored 0–0 correction . . . S-21

S10 Empirical shift and broadening analysis **S-22**

S11 Cartesian coordinates **S-25**
Final post-cycle revDSD-PBEP86-D4/def2-QZVP S_0 optimization coordinates retained for provenance . . . S-26
B3LYP/def2-TZVP S_0 minimum . . . S-26
B3LYP/def2-TZVP S_1 minimum used in the full-AH test . . . S-26
CAM-B3LYP/def2-TZVP S_0 minimum . . . S-27
CAM-B3LYP/def2-TZVP S_1 minimum used in the full-AH test . . . S-27
ωB97X-D4/def2-TZVP S_0 minimum . . . S-27
ωB97X-D4/def2-TZVP S_1 minimum used in the full-AH test . . . S-28

S1 Computational details and definitions

S1.1 Vertical excitation calculations

The common closed-shell S_0 geometry was optimized in the gas phase at the revDSD-PBEP86-D4/def2-QZVP level without imposing symmetry. Full linear-response TDDFT and TDA calculations were performed with ORCA 6.1.0 or 6.1.1 using B3LYP, CAM-B3LYP, R2SCAN-QIDH, ωB97M-D4, and ωB97X-D4. The basis-set sequence comprised def2-TZVP, def2-TZVPP, def2-QZVP, def2-QZVPPD, and aug-cc-pVQZ. Forty singlet roots were calculated, and the target state was followed through its energy order, weak oscillator strength, and dominant $n(\mathrm{N}) \rightarrow \pi^*(\mathrm{N{=}O})$ natural-transition-orbital pair.

For C, N, O, and F, ORCA generates the same orbital basis for def2-TZVP and def2-TZVPP. Both calculations are listed in the basis-resolved table, but they constitute one independent orbital-basis representation. Consequently, all basis spans and means use four distinct representations.

At a fixed geometry, the D4 term does not contribute directly to a linear-response excitation energy; the suffix is retained as part of the functional name. R2SCAN-QIDH and ωB97M-D4 depend explicitly on the kinetic-energy density τ. In time-dependent calculations, gauge invariance then requires the corresponding current-density contribution.[32] That restoration term is not present in the ORCA implementation used here, so these results are included in the complete comparison but are not used alone to rank absolute vertical accuracy. Grotjahn, Furche, and Kaupp showed that selected $n(\mathrm{N}) \rightarrow \pi^*(\mathrm{N{=}O})$ excitation energies can be strongly affected for some τ-dependent meta-GGAs.[33] The B3LYP, CAM-B3LYP, and ωB97X-D4 functionals used for the vibronic calculations do not depend on τ and are therefore unaffected by this specific current-density omission.

S1.2 Coupled-cluster and state-character calculations

Canonical EOM-CCSD calculations were performed at the authoritative common production geometry listed in Section S2.1 for ten singlet roots with converged right and left eigenvectors and exact transition-density matrices. The EOM-CC3/aug-cc-pVTZ calculation used the final post-cycle coordinates from the same unsymmetrized revDSD optimization (maximum Cartesian difference, 2.8621×10^{-5} Å); eT lists the atoms in a different order. The difference is physically negligible, but the coordinate strings remain distinct provenance lineages. The right and left excited-state vectors, biorthonormalization, and EOM transition moments converged for all three singlet roots.

Canonical RHF-CCSD(T)/cc-pVQZ calculations supplied the T_1, normalized Λ_1, and S diagnostics and the unrelaxed coupled-cluster density. Natural populations, bond indices, orbital occupancies, donor–acceptor interactions, and dipole moments were obtained with NBO 7.0. The occupation-constrained open-shell singlet ΔSCF calculation was carried out at revDSD-PBEP86-D4/def2-QZVP by exchanging the α HOMO and LUMO occupations and maintaining the imposed occupation by maximum overlap. Its unrelaxed MP2 electron density was used for the charge and bonding analysis, while atomic spin populations were obtained from the corresponding unrelaxed MP2 spin density.

State-averaged CAS(8,12)/aug-cc-pVQZ calculations used equal weights for ten singlet and ten triplet roots, followed by partially contracted NEVPT2 and quasi-degenerate spin–orbit coupling. CAS(8,7) and CAS(8,8)/def2-TZVP calculations followed the same SA-CASSCF(10,10) natural-orbital preparation protocol and strongly contracted NEVPT2, providing an active-space sensitivity test.

S1.3 Vibronic calculations

ORCA_ESD absorption calculations were performed at full TDDFT/def2-TZVP with B3LYP, CAM-B3LYP, and ωB97X-D4. In the vertical-gradient (VG) model, the S_1 gradient displaces the excited-state surface while the S_0 Hessian is retained. In the adiabatic-Hessian-after-a-step (AHAS) model, one step is taken toward the S_1 minimum and the excited-state Hessian is evaluated at the displaced geometry; Duschinsky rotation is included. Franck–Condon and first-order Herzberg–Teller intensities were calculated at 0 and 298.15 K.

The native spectra use homogeneous and inhomogeneous widths of 10 cm^{-1}. Figure 3 of the main manuscript uses a common Gaussian full width at half maximum (FWHM) of 80 cm^{-1}. The empirical shift/FWHM analysis described below tests only the similarity of the broad envelopes; it is not an electronic-energy correction.

S1.4 Observable definitions

$$E(\mathrm{eV}) = \frac{1239.841984}{\lambda(\mathrm{nm})}, \tag{1}$$

$$\widetilde{\nu}(\mathrm{cm}^{-1}) = \frac{10^7}{\lambda(\mathrm{nm})}. \tag{2}$$

E_{vert} is the electronic $S_1 - S_0$ gap evaluated at an S_0 geometry. E_{0-0} includes structural relaxation and the harmonic zero-point contribution within the specified model. The position of a 298 K band maximum additionally depends on vibronic intensity redistribution and broadening. Energy differences and rankings are therefore evaluated in eV or cm^{-1}, not in wavelength.

Table S1: Calculation and reporting conventions.

Item	Convention
Common S_0 geometry	revDSD-PBEP86-D4/def2-QZVP; gas phase; no imposed symmetry
TDDFT/TDA calculations	40 singlet roots; target identified by order, oscillator strength, and NTO character
Basis statistics	Four distinct orbital-basis representations; def2-TZVP and def2-TZVPP counted once
Experimental 0–0 origin	13929.7 cm^{-1} = 1.727063 eV = 717.89 nm
Strongest 298 K feature	14429.6 cm^{-1} = 1.789042 eV = 693.02 nm
Native ESD widths	10 cm^{-1} homogeneous and 10 cm^{-1} inhomogeneous
Main-text display width	80 cm^{-1} Gaussian FWHM

S2 Reference and method-specific structures

S2.1 Common S_0 production structure

```
6
CF3NO authoritative common production geometry; TDDFT/TDA and EOM-CCSD
C  -0.35828593858459   0.62087649909873   0.07190317341729
F   0.11740981759753  -0.00856657155861   1.13515957954301
N   0.78622367796591   0.71361370901691  -0.93351882552041
F  -1.41374014492081  -0.01462154477693  -0.39099067056343
F  -0.68411670953150   1.86328964004300   0.39312659805359
O   0.51674229747347   0.19368026817689  -1.97450385493005
```

Charge 0, multiplicity 1.

These exact coordinates were used by the production TDDFT/TDA and EOM-CCSD calculations and are retained in the current common-geometry templates. The final post-cycle coordinates used for ΔSCF and CC3 remain a distinct coordinate provenance, not a second exact common production geometry.

Table S2: Selected geometrical parameters of the common S_0 structure.

Parameter	Value
r(C–N) / Å	1.526
r(N=O) / Å	1.194
r(C–F) range / Å	1.316–1.324
∠C–N=O / degree	112.2
∠F_1–C–N / degree	106.8
∠F_2–C–N / degree	113.5
∠F_3–C–N / degree	106.7

S2.2 Effect of the S_0 geometry on the vertical energy

CAM-B3LYP/def2-TZVP full LR-TDDFT and TDA were evaluated at both the common revDSD-PBEP86-D4 geometry and a CAM-B3LYP/def2-TZVP S_0 minimum. The method-specific geometry raises the two vertical energies by 0.038 eV. This change is larger than the residual basis-set spans but substantially smaller than the paired TDA–full-LR shift.

Table S3: CAM-B3LYP/def2-TZVP geometry sensitivity.

Geometry	Formalism	E / eV	λ / nm	ΔE / eV	C–N / Å	N–O / Å	C–N–O / degree
Common revDSD S_0	full LR-TDDFT	1.742883	711.4	0.000000	1.526229	1.194404	112.241
Common revDSD S_0	TDA	1.858257	667.2	0.000000	1.526229	1.194404	112.241
CAM-B3LYP S_0	full LR-TDDFT	1.781169	696.1	+0.038286	1.524074	1.179204	112.607
CAM-B3LYP S_0	TDA	1.895856	654.0	+0.037599	1.524074	1.179204	112.607

These four single points reproduce the standardized vertical-benchmark protocol. The E_{vert} values retained in Section S8 are the values emitted by the separate accepted vibronic prerequisite branches and are not substituted into this protocol-matched geometry-sensitivity test.

S2.3 Local displacements used in the VG and AHAS models

The three functionals give a consistent local response: C–N contracts by 0.035–0.039 Å, N–O lengthens by 0.035–0.038 Å, and the C–N=O angle opens by 4.48–4.68°.

Table S4: Method-specific S_0 and locally displaced S_1 geometrical parameters used in the VG/AHAS calculations.

Functional	C–N$_0$ Å	C–N$_1$ Å	Δ(C–N) Å	N–O$_0$ Å	N–O$_1$ Å	Δ(N–O) Å	Δ∠CNO degree
CAM-B3LYP	1.524075	1.488915	-0.035159	1.179204	1.214497	+0.035293	+4.676
B3LYP	1.546004	1.506873	-0.039131	1.184254	1.222320	+0.038066	+4.476
ωB97X-D4	1.530550	1.495981	-0.034569	1.182423	1.217275	+0.034852	+4.506

S3 Experimental spectral data

The room-temperature profile was constructed from the 66 gas-phase band maxima reported by Mason[2] and distributed through the MPI-Mainz spectral atlas. The measured points, rather than an interpolated curve, define the experimental data. A shape-preserving interpolation is used only as a visual guide. The jet-cooled 0–0 origin is taken from the resolved spectroscopy literature. These quantities are kept distinct from one another and from the electronic vertical excitation.

Table S5: Experimental and coupled-cluster energy landmarks.

Quantity	cm^{-1}	eV	nm	Meaning
Jet-cooled 0–0 origin	13929.7	1.727063	717.89	Experimental origin
Strongest 298 K feature	14429.6	1.789042	693.02	Room-temperature band maximum
EOM-CC3 vertical energy	15920.7	1.973913	628.11	Electronic vertical reference
EOM-CCSD vertical range	16171–16180	2.005–2.006	618.4–618.0	Electronic vertical range

The strongest 298 K feature lies 499.9 cm^{-1} above the jet-cooled origin. The maximum digitized cross section is 9.01×10^{-20} cm^2 molecule^{-1}.

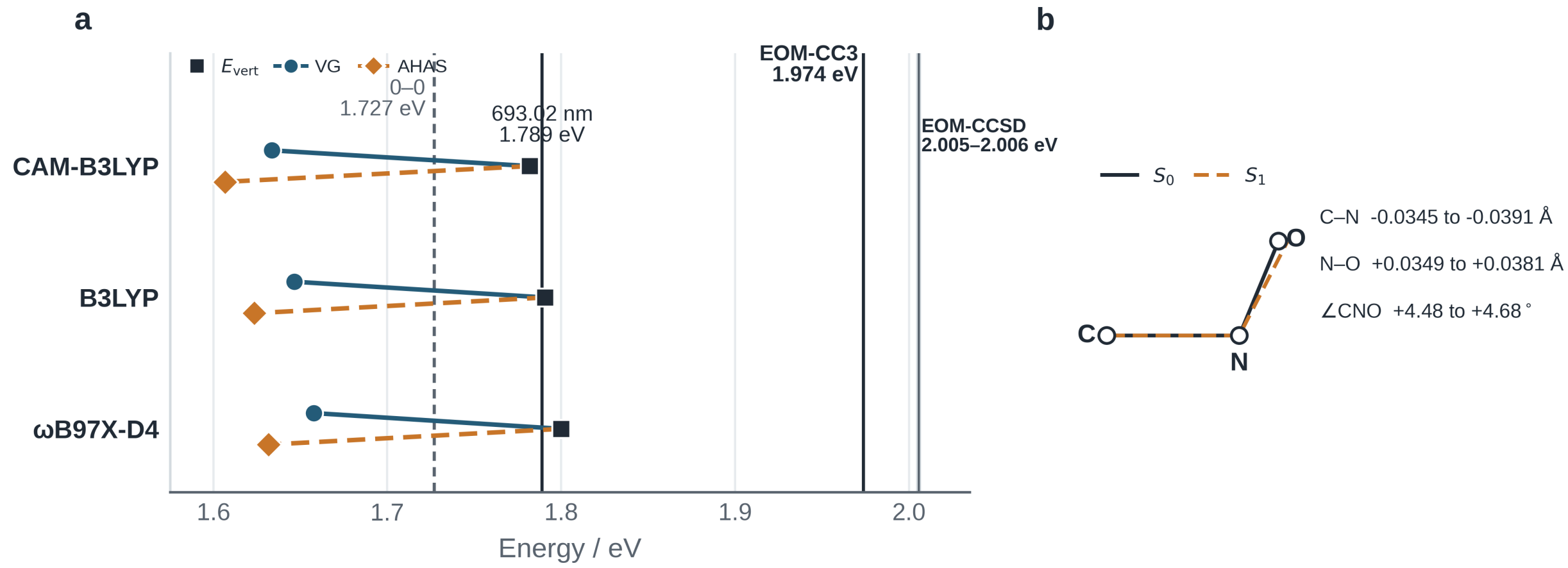

Figure S1: Method-specific vertical and harmonic 0–0 energies, together with the experimental 0–0 origin, the strongest 298 K feature, and the coupled-cluster vertical region. The structural diagram summarizes the local excited-state displacement used in the VG/AHAS calculations.

S4 Full LR-TDDFT and TDA vertical excitation results

Figure S2 summarizes the five functional pairs over the four distinct orbital-basis representations. The basis dependence is uniformly small: each TDDFT/TDA series spans less than 10 meV. By contrast, replacing full linear response by TDA raises the matched excitation by 0.102–0.179 eV.

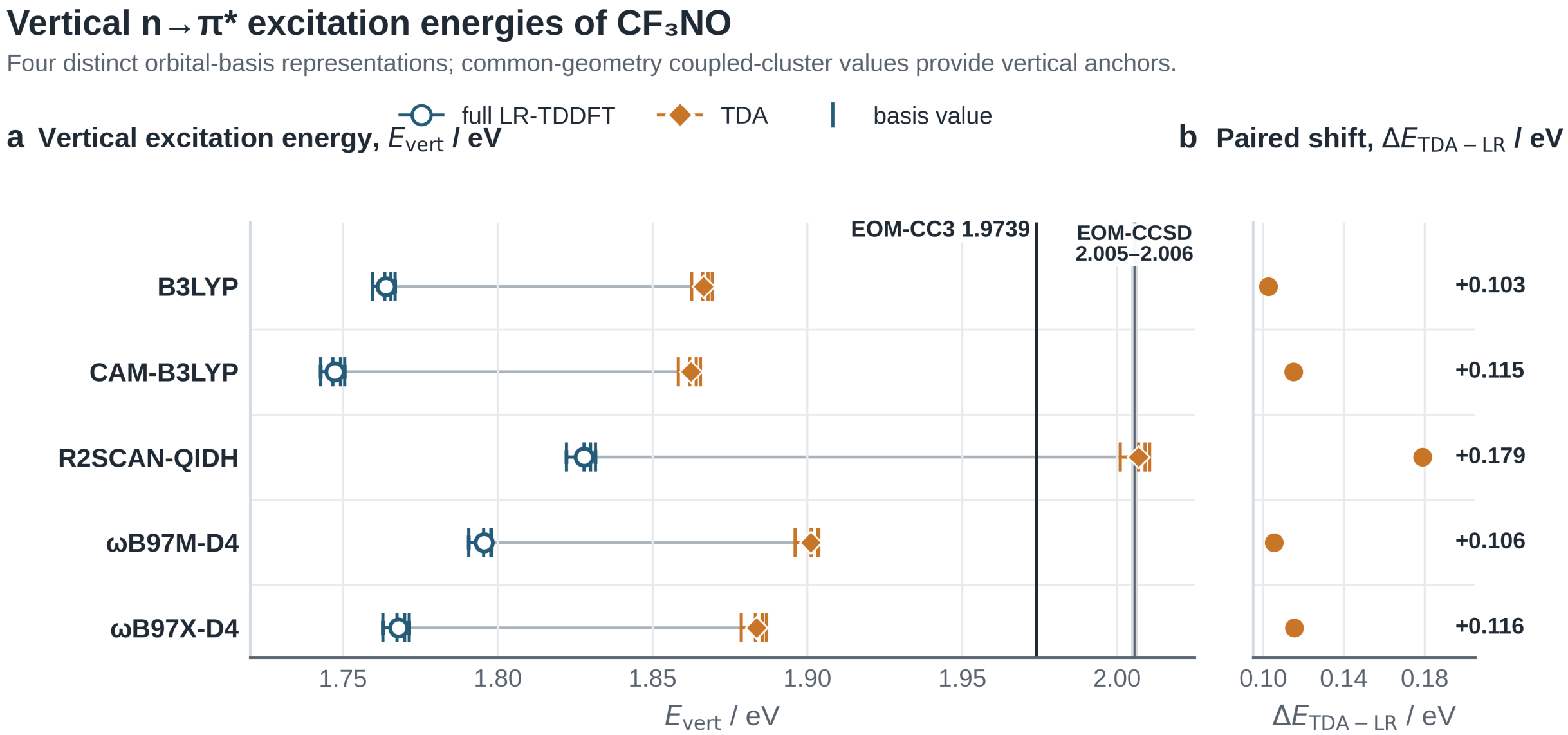

Figure S2: Full LR-TDDFT and TDA vertical excitation energies over the four distinct orbital-basis representations. Panel a shows the basis-resolved values and the EOM-CC3 and EOM-CCSD references; panel b gives the paired TDA–full-LR shift.

Table S6: Basis-resolved full LR-TDDFT and TDA results for the target $n(\mathrm{N}) \rightarrow \pi^*(\mathrm{N{=}O})$ state. f_L is the length-representation oscillator strength.

Functional	Formalism	Basis	E_{S_1} / eV	λ / nm	f_L
B3LYP	LR	aug-cc-pVQZ	1.766870	701.7	0.000160392
B3LYP	LR	def2-QZVP	1.763514	703.1	0.000162726
B3LYP	LR	def2-QZVPPD	1.765460	702.3	0.000159474
B3LYP	LR	def2-TZVP	1.759598	704.6	0.000177353
B3LYP	LR	def2-TZVPP†	1.759598	704.6	0.000177353
CAM-B3LYP	LR	aug-cc-pVQZ	1.750626	708.2	0.000187003
CAM-B3LYP	LR	def2-QZVP	1.746813	709.8	0.000189136
CAM-B3LYP	LR	def2-QZVPPD	1.749227	708.8	0.000186087
CAM-B3LYP	LR	def2-TZVP	1.742883	711.4	0.000206549
CAM-B3LYP	LR	def2-TZVPP†	1.742883	711.4	0.000206549
R2SCAN-QIDH	LR	aug-cc-pVQZ	1.831492	677.0	0.000216244
R2SCAN-QIDH	LR	def2-QZVP	1.827842	678.3	0.000216769
R2SCAN-QIDH	LR	def2-QZVPPD	1.829898	677.5	0.000216297
R2SCAN-QIDH	LR	def2-TZVP	1.822137	680.4	0.000233041
R2SCAN-QIDH	LR	def2-TZVPP†	1.822137	680.4	0.000233041
ωB97M-D4	LR	aug-cc-pVQZ	1.798071	689.5	0.000196294
ωB97M-D4	LR	def2-QZVP	1.795475	690.5	0.000197925
ωB97M-D4	LR	def2-QZVPPD	1.797813	689.6	0.00019512
ωB97M-D4	LR	def2-TZVP	1.790694	692.4	0.000217972
ωB97M-D4	LR	def2-TZVPP†	1.790694	692.4	0.000217972
ωB97X-D4	LR	aug-cc-pVQZ	1.771388	699.9	0.000191277
ωB97X-D4	LR	def2-QZVP	1.767512	701.5	0.000192733
ωB97X-D4	LR	def2-QZVPPD	1.769924	700.5	0.000190411
ωB97X-D4	LR	def2-TZVP	1.762962	703.3	0.00020857
ωB97X-D4	LR	def2-TZVPP†	1.762962	703.3	0.00020857

Table S6: Basis-resolved full LR-TDDFT and TDA results. (continued)

Functional	Formalism	Basis	E_{S_1} / eV	λ / nm	f_L
B3LYP	TDA	aug-cc-pVQZ	1.869229	663.3	0.000358421
B3LYP	TDA	def2-QZVP	1.866240	664.4	0.000360162
B3LYP	TDA	def2-QZVPPD	1.867879	663.8	0.000357045
B3LYP	TDA	def2-TZVP	1.862589	665.7	0.000380501
B3LYP	TDA	def2-TZVPP†	1.862589	665.7	0.000380501
CAM-B3LYP	TDA	aug-cc-pVQZ	1.865381	664.7	0.000430586
CAM-B3LYP	TDA	def2-QZVP	1.861935	665.9	0.00043255
CAM-B3LYP	TDA	def2-QZVPPD	1.864091	665.1	0.000429213
CAM-B3LYP	TDA	def2-TZVP	1.858257	667.2	0.000457497
CAM-B3LYP	TDA	def2-TZVPP†	1.858257	667.2	0.000457497
R2SCAN-QIDH	TDA	aug-cc-pVQZ	2.010509	616.7	0.000518805
R2SCAN-QIDH	TDA	def2-QZVP	2.006968	617.8	0.000519301
R2SCAN-QIDH	TDA	def2-QZVPPD	2.008954	617.2	0.000518895
R2SCAN-QIDH	TDA	def2-TZVP	2.000983	619.6	0.000542877
R2SCAN-QIDH	TDA	def2-TZVPP†	2.000983	619.6	0.000542877
ωB97M-D4	TDA	aug-cc-pVQZ	1.903647	651.3	0.00043686
ωB97M-D4	TDA	def2-QZVP	1.901144	652.2	0.000437346
ωB97M-D4	TDA	def2-QZVPPD	1.903368	651.4	0.000434805
ωB97M-D4	TDA	def2-TZVP	1.896039	653.9	0.000463991
ωB97M-D4	TDA	def2-TZVPP†	1.896039	653.9	0.000463991
ωB97X-D4	TDA	aug-cc-pVQZ	1.886800	657.1	0.00043488
ωB97X-D4	TDA	def2-QZVP	1.883171	658.4	0.000435744
ωB97X-D4	TDA	def2-QZVPPD	1.885399	657.6	0.00043348
ωB97X-D4	TDA	def2-TZVP	1.878622	660.0	0.000457359
ωB97X-D4	TDA	def2-TZVPP†	1.878622	660.0	0.000457359

Note: †def2-TZVPP and def2-TZVP generate the same orbital basis for the atoms in CF_3NO; only one contributes to the four-representation statistics.

Table S7: Energy ranges over the four distinct orbital-basis representations. ΔE is signed relative to EOM-CC3 (1.973912572824 eV): negative values are red-shifted and positive values are blue-shifted.

Method	Formalism	E_{vert} / eV	Span / meV	ΔE vs CC3 / eV	$\Delta E_{\text{TDA-LR}}$ / eV
B3LYP	full LR	1.760–1.767	7.3	−0.214 to −0.207	—
B3LYP	TDA	1.863–1.869	6.6	−0.111 to −0.105	+0.102 to +0.103
CAM-B3LYP	full LR	1.743–1.751	7.7	−0.231 to −0.223	—
CAM-B3LYP	TDA	1.858–1.865	7.1	−0.116 to −0.109	+0.115
R2SCAN-QIDH	full LR	1.822–1.831	9.4	−0.152 to −0.142	—
R2SCAN-QIDH	TDA	2.001–2.011	9.5	+0.027 to +0.037	+0.179
ωB97M-D4	full LR	1.791–1.798	7.4	−0.183 to −0.176	—
ωB97M-D4	TDA	1.896–1.904	7.6	−0.078 to −0.070	+0.105 to +0.106
ωB97X-D4	full LR	1.763–1.771	8.4	−0.211 to −0.203	—
ωB97X-D4	TDA	1.879–1.887	8.2	−0.095 to −0.087	+0.115 to +0.116
EOM-CCSD	EOM	2.005–2.006	1.2	+0.031 to +0.032	—
EOM-CC3	EOM	1.973913	—	reference	—

All full-LR values are red-shifted relative to EOM-CC3. TDA moves every functional toward the coupled-cluster region: R2SCAN-QIDH becomes slightly blue-shifted, whereas the other four remain red-shifted. The target root retains the same weak $n(\mathrm{N}) \rightarrow \pi^*(\mathrm{N{=}O})$ character throughout the sequence.

S5 Coupled-cluster results

S5.1 EOM-CCSD

The canonical EOM-CCSD excitation energy varies by only 1.2 meV over the four distinct orbital-basis representations. Right and left length-representation oscillator strengths differ by approximately 8–9%, and the target root contains 93.39–93.43% singles character.

Table S8: Basis-resolved canonical EOM-CCSD results. f_R and f_L are the right- and left-transition-density length-representation oscillator strengths; f_{avg} is their geometric mean, $f_{\mathrm{avg}} = \sqrt{f_R f_L}$.

Basis	E / eV	λ / nm	f_R	f_L	f_{avg}	Singles / %
def2-TZVP	2.005057	618.4	0.000315177	0.000339881	0.000327296	93.39
def2-TZVPP†	2.005057	618.4	0.000315177	0.000339881	0.000327296	93.39
def2-QZVP	2.005008	618.4	0.000297999	0.000322093	0.000309812	93.43
def2-QZVPPD	2.005787	618.1	0.000294896	0.000319746	0.000307070	93.41
aug-cc-pVQZ	2.006186	618.0	0.000294757	0.000320603	0.000307408	93.42

S5.2 EOM-CC3

The right- and left-hand nonlinear Davidson calculations converged for three singlet roots, followed by biorthonormalization and evaluation of the EOM transition moments. For S_1, the converged right- and left-hand energies are 1.973912572824 and 1.973858432870 eV, and the corresponding eigenvectors contain 96.5021% and 92.9779% singles character. Its final oscillator strengths are $2.53361913 \times 10^{-4}$ in the length gauge, $2.47092916 \times 10^{-4}$ in the velocity gauge, and $2.50343070 \times 10^{-4}$ in the mixed gauge, consistent with the weakly allowed character of the transition. The CC3 calculation uses aug-cc-pVTZ, whereas the EOM-CCSD sequence uses the bases listed above; their 0.031–0.032 eV separation is therefore not a strictly matched-basis triples increment. The very small EOM-CCSD basis span nevertheless indicates that a large residual basis effect is unlikely.

Table S9: Converged right-hand EOM-CC3/aug-cc-pVTZ singlet roots at the post-cycle revDSD-PBEP86-D4/def2-QZVP S_0 geometry. $|R_1|/|R|$ is the singles fraction reported by eT. Orbital 24 is the HOMO and virtual orbital 1 is the LUMO.

State	Energy / E_h	Energy / eV	cm^{-1}	λ / nm	$\lvert R_1\rvert/\lvert R\rvert$ / %	Dominant component
S_1	0.072539949688	1.973912572824	15920.7	628.1139	96.5021	HOMO (24)→LUMO (1), $R = +0.919454$; target $n(\mathrm{N}) \to \pi^*(\mathrm{N{=}O})$ state
S_2	0.213530468660	5.810460009741	46864.5	213.3810	6.9318	dominant double-excitation component $(1, 24; 1, 24)$, $R = +0.828625$
S_3	0.252105204854	6.860132046945	55330.7	180.7315	87.5950	largest single component occupied 22→virtual 1, $R = -0.572502$

Table S10: Final EOM-CC3/aug-cc-pVTZ transition properties after convergence of the right and left excited-state vectors. f_{len}, f_{vel}, and f_{mix} are the length-, velocity-, and mixed-gauge oscillator strengths printed by eT.

State	Energy / eV	$\lvert R_1\rvert/\lvert R\rvert$ / %	$\lvert L_1\rvert/\lvert L\rvert$ / %	f_{len}	f_{vel}	f_{mix}
S_1	1.973912572824	96.5021	92.9779	$2.53361913 \times 10^{-4}$	$2.47092916 \times 10^{-4}$	$2.50343070 \times 10^{-4}$
S_2	5.810460009741	6.9318	3.8558	7.8907627×10^{-5}	$1.00403741 \times 10^{-4}$	8.3788772×10^{-5}
S_3	6.860132046945	87.5950	84.9052	$2.448565602 \times 10^{-3}$	$2.392806563 \times 10^{-3}$	$2.421147586 \times 10^{-3}$

Table S11: Principal computational parameters of the EOM-CC3 calculation.

Quantity	Value
Program and method	eT 2.5.1; RHF/CC3 response
Basis set	aug-cc-pVTZ
Requested roots	Three singlet states
Atomic and molecular orbitals	276 AOs; 276 MOs
Orbital partition	24 occupied; 252 virtual
Cholesky decomposition	threshold 10^{-4}; 1225 vectors
RHF total energy	$-465.586696896802\ E_h$
CC3 ground-state energy	$-467.092669145887\ E_h$
Convergence	Ground-state amplitudes, multipliers, all three right and left excited-state vectors, biorthonormalization, and EOM transition moments converged

S6 Ground-state and ΔSCF descriptors

Table S12: Ground-state coupled-cluster and electronic-structure descriptors.

Quantity	Value	Interpretation or source
E(CCSD(T))	$-467.101303715\ E_h$	RHF-CCSD(T)/cc-pVQZ
T_1 diagnostic	0.01344	reference-state singles diagnostic
Normalized Λ_1 singles norm	0.01069	complementary singles diagnostic
S diagnostic	0.690097	RHF reference diagnostic
Largest eigenvalue used in S	0.031002	auxiliary quantity
$\sqrt{\lambda_{\mathrm{max}}}$	0.176074	auxiliary quantity
NPA charge C / e	+1.071	revDSD S_0 density
NPA charge N / e	+0.100	revDSD S_0 density
NPA charge O / e	−0.206	revDSD S_0 density
NPA charge CF_3 / NO	+0.106 / −0.106	fragment sums
Dipole moment	0.268 D	revDSD S_0 density
NPA charge C, SCF → CCSD(T)	+1.113 → +0.946	reduced polarization after correlation
Mayer C–N bond order, SCF → CCSD(T)	1.035 → 0.967	RHF and Λ-unrelaxed CCSD(T) densities
Mayer N–O bond order, SCF → CCSD(T)	1.920 → 1.705	RHF and Λ-unrelaxed CCSD(T) densities
π/π^*(N=O) occupancy	1.990/0.008 → 1.885/0.113	SCF to unrelaxed CCSD(T) density

The T_1, Λ_1, and S values assess the equilibrium S_0 reference; they are not state-specific measures of an EOM excitation energy.

Table S13: Vertical occupation-constrained ΔSCF descriptors. Charges and Mayer bond orders were obtained from the unrelaxed MP2 electron densities. Atomic spin populations for the imposed open-shell singlet configuration were obtained from the corresponding unrelaxed MP2 spin density.

Descriptor	S_0	$S_{1,\mathrm{vert}}$	Change or interpretation
$q_{\mathrm{NPA}}(\mathrm{N})$ / e	+0.100	−0.061	N gains 0.161 e
$q_{\mathrm{NPA}}(\mathrm{O})$ / e	−0.206	−0.105	O becomes 0.101 e less negative
$q_{\mathrm{NPA}}(\mathrm{CF_3})$ / e	+0.106	+0.166	CF_3 loses 0.060 e
Spin population(N)	0.000	+0.589	localized α excess on N
Spin population(O)	0.000	−0.429	localized β excess on O
Mayer BO(C–N)	0.935	0.911	small vertical weakening
Mayer BO(N=O)	1.751	1.507	pronounced π^* antibonding response
Dipole / D	0.268	0.351	increase of 0.083 D

To use a single bond-index definition, Mayer bond orders are reported in both analyses. The correlated ground-state values refer to the RHF and Λ-unrelaxed CCSD(T)/cc-pVQZ electron densities, whereas the vertical S_0/S_1 values refer to the revDSD-PBEP86-D4 unrelaxed MP2 electron densities. Only changes within each internally consistent pair are interpreted; absolute bond-order values are not compared between the two electronic-structure analyses. For the open-shell S_1 analysis, atomic spin populations were obtained from the corresponding unrelaxed MP2 spin density. The density gain at nitrogen is consistent with the substantial N contribution to the π^*(N=O) acceptor. Because the ΔSCF result is a self-consistent difference between two total densities, it also includes orbital relaxation; it is not an atomic partition of a single promoted electron.

S7 CASSCF/NEVPT2 and spin–orbit analysis

Table S14: CAS(8,12) active-orbital composition. Active orbitals correspond to ORCA molecular orbitals 20–31.

Active orbital	ORCA MO	NOON	Leading atomic contributions / %	Qualitative role
1	20	1.973	N 42.8, C 21.3, O 4.8	occupied N/C valence support
2	21	1.843	O 48.3, N 29.2	occupied nitroso valence support
3	22	1.824	N 39.5, O 37.4	occupied nitroso valence support
4	23	1.062	O 37.7, N 30.4, C 6.3	frontier nitroso n/π orbital
5	24	0.659	N 35.1, O 29.5	frontier nitroso π^* orbital
6	25	0.201	C 35.2, N 32.3, O 14.2	low-occupation valence acceptor
7	26	0.102	C 43.5, N 26.4, O 14.7	valence polarization
8	27	0.101	N 34.9, C 25.4, O 22.4	N/O/C polarization
9	28	0.101	C 49.7, N 11.3, O 9.2	C-centered polarization
10	29	0.100	O 29.9, N 27.3, C 21.6	nitroso/C polarization
11	30	0.028	N 41.5, C 22.2	high-virtual nitroso/C support
12	31	0.006	N 55.2, O 14.2	high-virtual nitroso support

Table S15: Dominant CAS(8,12) configurations and state-isolation diagnostics.

Quantity	Value	Interpretation
S_0 leading-configuration weight	0.897	largest secondary weight 0.048
S_1 leading $n(\text{N}) \rightarrow \pi^*(\text{N=O})$ weight	0.883	largest secondary weight 0.045
PC-NEVPT2 T_1	1.111 eV	lowest triplet in the diagnostic manifold
PC-NEVPT2 S_1	2.009 eV	target singlet
PC-NEVPT2 S_2	4.843 eV	next singlet in the diagnostic manifold
$S_2 - S_1$	2.834 eV	isolated target within the singlet manifold
$S_1 - T_1$	0.899 eV	substantial singlet–triplet separation
QDPT state correlated with S_1	99.9997% singlet	negligible spin–orbit mixing

Table S16: Active-space sensitivity at def2-TZVP with strongly contracted NEVPT2.

Active space	T_1 / eV	S_1 / eV	S_2 / eV	$S_1 - T_1$ / eV	Calculation
CAS(8,7)	1.097	1.982	4.884	0.885	SA-CASSCF(10,10) natural-orbital preparation
CAS(8,8)	1.121	2.000	4.922	0.879	independent calculation using the same preparation protocol

The CAS(8,7) and CAS(8,8) calculations test the stability of the low-energy pattern with respect to the number of active orbitals. They complement, rather than replace, the larger CAS(8,12)/aug-cc-pVQZ calculation.

S8 VG and AHAS vibronic calculations

The method-specific vertical energies are 1.781903 eV (CAM-B3LYP), 1.790748 eV (B3LYP), and 1.800012 eV (ωB97X-D4). The VG and AHAS harmonic 0–0 energies span 1.6066–1.6577 eV, placing them 0.069–0.120 eV below the experimental jet-cooled origin. At 298 K, the global maxima of the native fine spectra remain close to the respective calculated origins.

Table S17: VG/AHAS vibronic results. E_{max}^{298} is the global maximum of the native fine-resolution 298 K FC+HT trace. The HT fraction is integrated over 1.4–2.2 eV.

Functional	Model	E_{vert} eV	E_{0-0} eV	$E_{\mathrm{vert}} - E_{0-0}$ eV	E_{max}^{298} eV	ΔE_{0-0} vs exp. eV	HT / %
CAM-B3LYP	VG	1.781903	1.633580	0.148323	1.633658	-0.093483	13.80
CAM-B3LYP	AHAS	1.781903	1.606634	0.175269	1.606302	-0.120428	14.71
B3LYP	VG	1.790748	1.646562	0.144186	1.646481	-0.080500	12.70
B3LYP	AHAS	1.790748	1.623433	0.167315	1.623400	-0.103630	13.93
ωB97X-D4	VG	1.800012	1.657720	0.142292	1.657595	-0.069343	13.35
ωB97X-D4	AHAS	1.800012	1.631620	0.168392	1.631094	-0.095443	18.39

Table S18: VG/AHAS normal-mode mapping and reorganization descriptors.

Functional	Model	RMSD / Å	$\langle\lvert J_{\mathrm{max}}\rvert\rangle$	Diagonality	Reorganization / cm^{-1}
CAM-B3LYP	VG	0.029641	1.000000	1.000000	420.50
CAM-B3LYP	AHAS	0.029641	0.905522	0.914459	420.51
B3LYP	VG	0.028766	1.000000	1.000000	444.07
B3LYP	AHAS	0.028766	0.877081	0.873442	444.07
ωB97X-D4	VG	0.028650	1.000000	1.000000	419.02
ωB97X-D4	AHAS	0.028673	0.910815	0.919813	419.00

Approximately four-fifths of the calculated reorganization energy is associated with the N–O stretching coordinate near 1710–1770 cm^{-1}. The experimental spectrum instead develops substantial intensity through lower-frequency structure, including intervals near 450–500 and 700–800 cm^{-1} and a hindered-torsional progression in the jet-cooled spectrum.

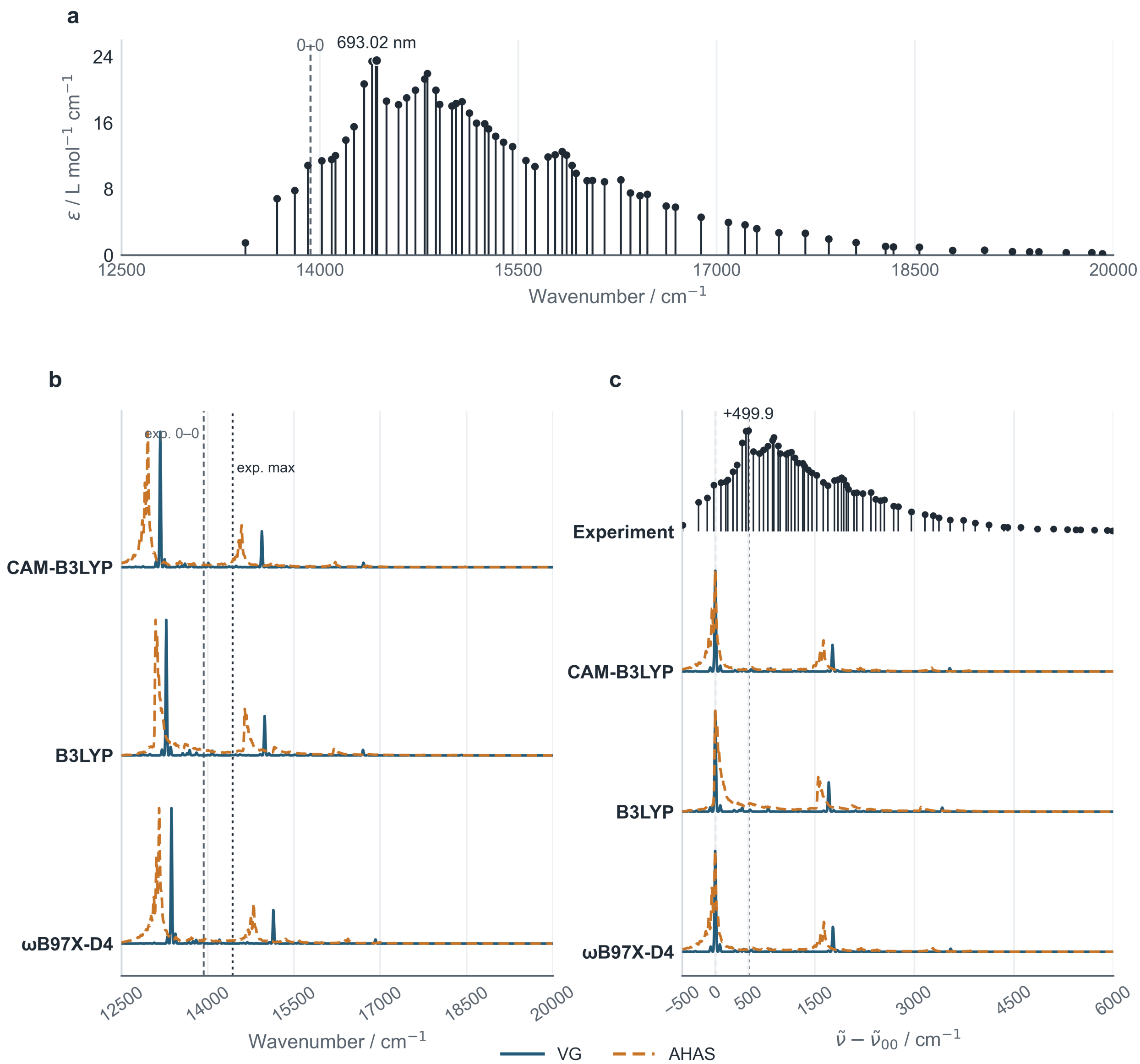

Figure S3: Native fine-resolution 298 K VG and AHAS FC+HT spectra. Panel a shows the 66 experimental band maxima from Mason[2]; panel b gives the spectra on an absolute energy scale; panel c references each trace to its own 0–0 energy.

S9 Fully optimized adiabatic-Hessian analysis

The fully optimized adiabatic-Hessian (full-AH) construction pairs independently optimized S_0 and S_1 minima and their Hessians. The stationary points contain no imaginary modes. The limitation arises instead from the distance between the minima: the aligned S_0/S_1

RMSD is approximately 0.28 Å, nearly ten times the local VG/AHAS displacement, and the Duschinsky diagonality falls to 0.647–0.658.

Table S19: Full-AH mapping and spectral diagnostics. Fine and broad columns give the global 298 K maximum at the corresponding resolution. The ωB97X-D4 HT calculation was not continued after the same poor mapping regime had been established.

Functional	RMSD / Å	J_{max}	Diagonality	Reorg. / cm^{-1}	E_{0-0} / eV	Fine max / eV	Broad max / eV
B3LYP	0.280618	0.758255	0.647413	873.32	1.506666	1.507137	2.179066
CAM-B3LYP	0.279479	0.770079	0.657661	768.59	1.492129	1.491750	2.188469
ωB97X-D4	0.281096	0.773370	0.658333	733.18	1.522161	—	1.815626

S9.1 Exploratory scalar CC3-anchored 0–0 correction

The failure of the full-AH spectral mapping does not prevent a separate scalar use of the independently optimized stationary-point energies and harmonic zero-point vibrational energies (ZPVEs). We therefore evaluated an exploratory CC3-anchored 0–0 correction without using the full-AH Duschinsky matrices, intensities, peak positions, or band shapes. For each functional,

$$E_{0-0}^{\mathrm{DFT}} = \left[E_{S_1}^{\mathrm{min}} - E_{S_0}^{\mathrm{min}} + \mathrm{ZPVE}_{S_1} - \mathrm{ZPVE}_{S_0}\right] \times 27.211386245988 \text{ eV}. \tag{3}$$

Two DFT vertical-energy geometry conventions were then retained explicitly:

$$E_{0-0}^{\mathrm{A}} = E_{\mathrm{vert}}^{\mathrm{CC3,post\text{-}cycle}} + \left(E_{0-0}^{\mathrm{DFT}} - E_{\mathrm{vert}}^{\mathrm{DFT,common}}\right), \tag{4}$$

$$E_{0-0}^{\mathrm{B}} = E_{\mathrm{vert}}^{\mathrm{CC3,post\text{-}cycle}} + \left(E_{0-0}^{\mathrm{DFT}} - E_{\mathrm{vert}}^{\mathrm{DFT,method\text{-}specific}}\right). \tag{5}$$

Here $E_{\mathrm{vert}}^{\mathrm{CC3,post\text{-}cycle}} = 1.973912573$ eV is the CC3 anchor at the post-cycle, near-common revDSD geometry, not at the exact common geometry; the experimental jet-cooled origin is 1.727063 eV. Protocol A uses the common-geometry DFT correction; Protocol B uses its method-specific S_0 counterpart. The harmonized CAM-B3LYP method-specific vertical value, 1.781169 eV, differs from the earlier ESD value by only −0.000734 eV and is retained

as the current standardized result.

Table S20: Exploratory scalar CC3-anchored 0–0 corrections (eV). Δ_{exp} is the signed deviation from the 1.727063 eV jet-cooled origin, and the final column is the absolute difference between Protocols A and B.

Functional	E_{0-0}^{DFT}	$E_{\text{vert}}^{\text{DFT,common}}$	$E_{\text{vert}}^{\text{DFT,method}}$	A	Δ_{exp}(A)	B	Δ_{exp}(B)	$\lvert A-B\rvert$
B3LYP	1.506627	1.759598	1.790748	1.720942	-0.006121	1.689792	-0.037271	0.031150
CAM-B3LYP	1.492156	1.742883	1.781169	1.723185	-0.003878	1.684899	-0.042164	0.038286
ωB97X-D4	1.522128	1.762962	1.800012	1.733078	+0.006015	1.696029	-0.031034	0.037050

Protocol A gives 1.720942–1.733078 eV, with signed deviations of −0.006121 to +0.006015 eV from the experimental origin. Protocol B gives 1.684899–1.696029 eV and remains lower by 0.031034–0.042164 eV. Most importantly, changing the vertical-energy geometry convention shifts an individual composite result by 0.031150–0.038286 eV. The close Protocol A values are therefore an exploratory scalar consistency check only. They neither validate the failed full-AH spectral construction nor support a functional ranking, and they are not used as the primary spectral benchmark.

For B3LYP and CAM-B3LYP, broadening moves the 298 K global maximum from approximately 1.49–1.51 eV to 2.18–2.19 eV. This peak inversion shows that the full-AH envelope is not stable with respect to linewidth. The ωB97X-D4 FC result occupies the same poor-mapping regime. The full-AH spectra are therefore not used in the physical interpretation of the band.

S10 Empirical shift and broadening analysis

For each of nine 298 K source traces (three functionals and VG, AHAS, or full AH), a constant energy shift and an effective Gaussian FWHM were scanned. The shift interval was −0.8 to +0.8 eV, using 0.004 eV coarse steps and 0.0005 eV refinement. The FWHM interval extended to 2000 cm^{-1}, using 20 cm^{-1} coarse steps and 2.5 cm^{-1} refinement. Fits used the 66 measured band maxima from Mason[2] after peak normalization.

The composite score was

$$S = 0.50r + 0.30O + 0.20(1 - \mathrm{RMSE}), \tag{6}$$

where r is the Pearson correlation coefficient and O is the normalized overlap. This score is a comparative plotting metric, not a likelihood or a physical model-selection criterion.

Table S21: Best constant-shift and Gaussian-FWHM parameters. These are post-processing sensitivity parameters, not corrected spectral predictions.

Functional	Model	Shift / eV	FWHM / cm^{-1}	r	RMSE	Overlap
ωB97X-D4	AHAS	+0.1865	1270.0	0.9788	0.0736	0.8911
CAM-B3LYP	AHAS	+0.2115	1275.0	0.9790	0.0735	0.8898
B3LYP	AHAS	+0.1690	1152.5	0.9797	0.0723	0.8859
B3LYP	VG	+0.1675	1405.0	0.9776	0.0789	0.8806
CAM-B3LYP	VG	+0.1845	1467.5	0.9775	0.0825	0.8730
ωB97X-D4	VG	+0.1620	1497.5	0.9774	0.0849	0.8690
ωB97X-D4	AH	-0.1740	80.0	0.8652	0.5499	0.3388
CAM-B3LYP	AH	-0.3485	275.0	0.8757	0.6077	0.3163
B3LYP	AH	-0.3295	62.5	0.8615	0.5976	0.3201

The VG and AHAS profiles require positive shifts of +0.1620 to +0.2115 eV and broad effective widths of 1152.5–1497.5 cm^{-1} to reproduce the gross envelope. Their best correlations are 0.977–0.980. The full-AH profiles remain poor after optimization, with normalized RMSE values of 0.550–0.608 and overlaps of only 0.32–0.34.

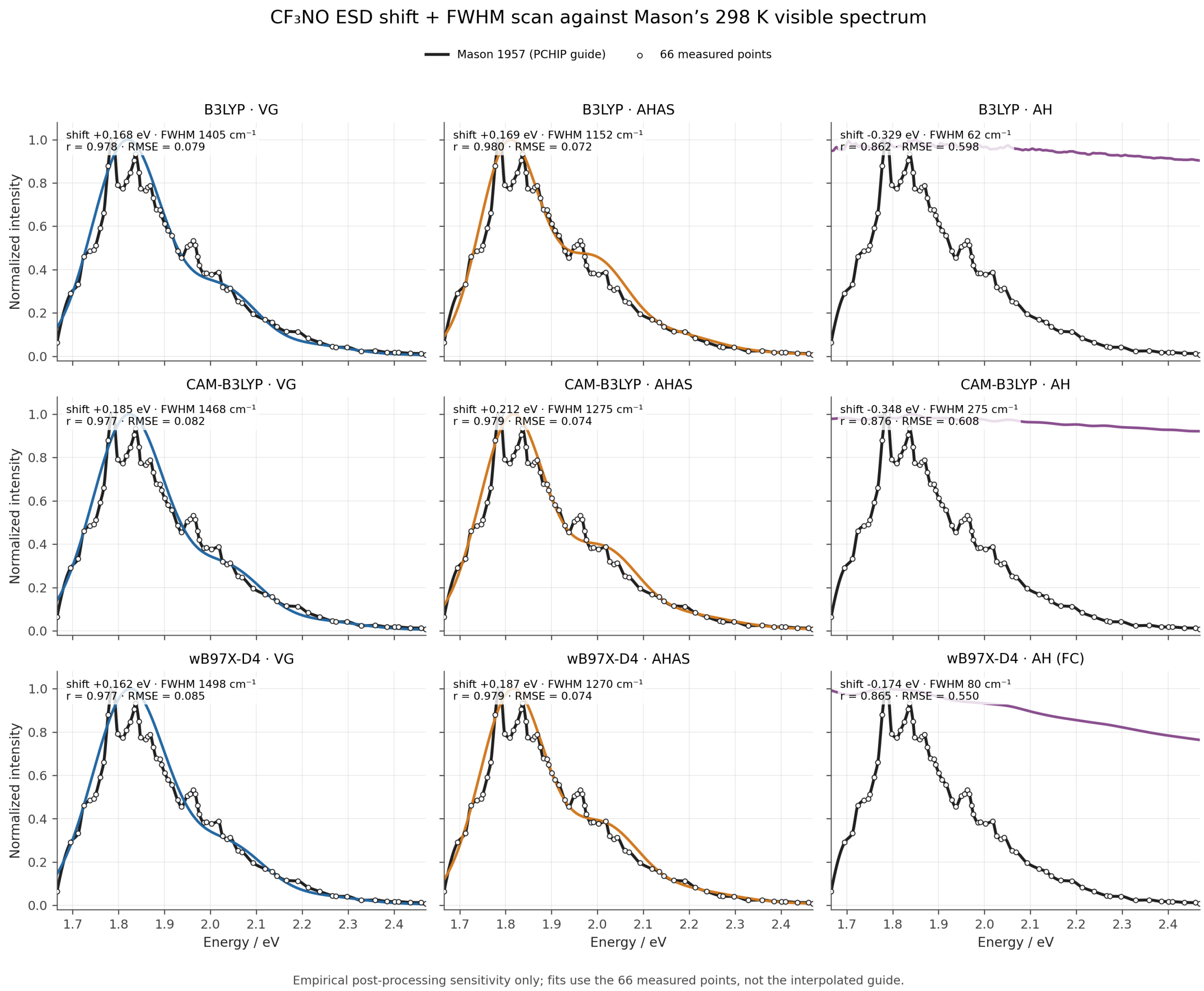

Figure S4: Best shift/FWHM overlays for the nine 298 K traces against the 66 experimental band maxima from Mason[2]. VG and AHAS recover the broad envelope only after substantial shifting and broadening; the full-AH profiles retain an incorrect intensity distribution.

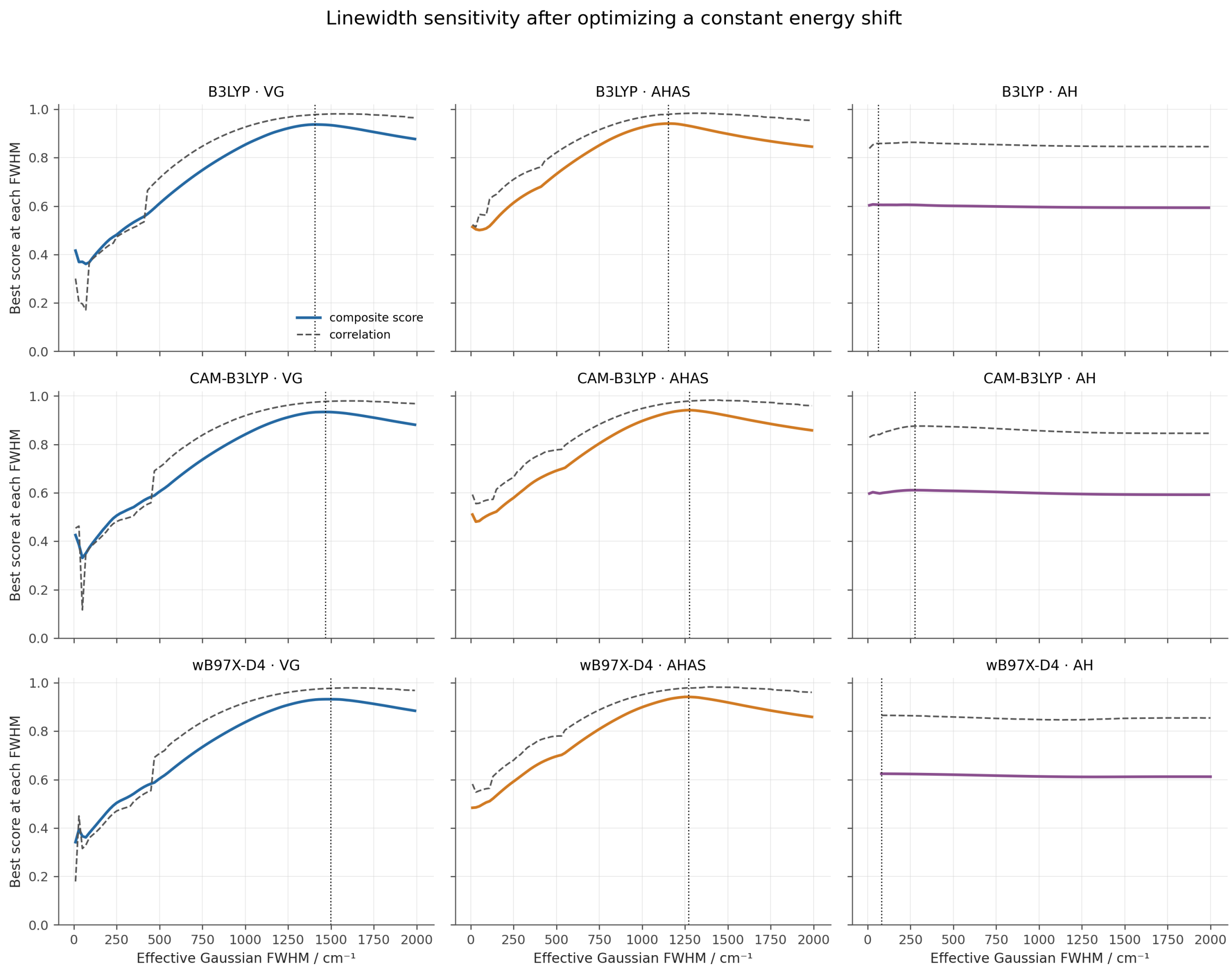

Figure S5: Linewidth sensitivity after optimizing the constant shift at each FWHM. The VG/AHAS scores improve only after broadening to approximately 1150–1500 cm^{-1}, whereas the full-AH scores remain low.

S11 Cartesian coordinates

The method-specific S_0 and S_1 minima, together with the final post-cycle revDSD-PBEP86-D4/def2-QZVP optimization coordinates retained for provenance, are listed below in Å. The authoritative common-production coordinates are given in Section S2.1. The optimized S_1 structures belong to the full-AH test; the AHAS calculations use the local displaced structures summarized in Table S4.

Final post-cycle revDSD-PBEP86-D4/def2-QZVP S_0 optimization coordinates retained for provenance

```
6
CF3NO revDSD-PBEP86-D4/def2-QZVP S0 minimum
C  -0.35828868  0.62088219  0.07190034
F   0.11741602 -0.00857731  1.13514287
N   0.78621135  0.71364018 -0.93352896
F  -1.41375262 -0.01461461 -0.39098488
F  -0.68411733  1.86328990  0.39313960
O   0.51676425  0.19365165 -1.97449297
```

These coordinates were used for the ΔSCF and CC3 calculations; they are not the authoritative TDDFT/TDA/EOM-CCSD common-production coordinates reported in Section S2.1.

B3LYP/def2-TZVP S_0 minimum

```
6
CF3NO B3LYP/def2-TZVP S0 minimum; E = -467.538739092171 Eh
C  -0.36389719890140  0.62016650689158  0.07525034703744
F   0.11941451123749 -0.01110235037016  1.14118046742550
N   0.79209152229431  0.71155641267768 -0.94722966658839
F  -1.42667074234372 -0.01722191813511 -0.38503527717439
F  -0.68664528176016  1.86943007148891  0.39737107374075
O   0.52994017947348  0.19544327744711 -1.98036094444091
```

B3LYP/def2-TZVP S_1 minimum used in the full-AH test

```
6
CF3NO B3LYP/def2-TZVP S1 minimum; E = -467.482795795688 Eh
C  -0.34329186000981  0.55327205847978  0.08655550253851
F   0.09051601244507  0.10292087700397  1.25072788593494
N   0.69797111134609  0.31363436561704 -0.89959412549857
F  -1.47337442837700 -0.07993254898912 -0.24662318313670
F  -0.61859132872121  1.85854291108635  0.18399616495499
O   0.61100449331686  0.61983433680197 -2.07388624479316
```

CAM-B3LYP/def2-TZVP S_0 minimum

```
6
CF3NO CAM-B3LYP/def2-TZVP S0 minimum; E = -467.598888964875 Eh
C  -0.35866734773712  0.62055604445280  0.07071804123243
F   0.11901894119018 -0.00816770868557  1.13171576518565
N   0.78128349618710  0.71075395293583 -0.93684251072480
F  -1.41382076456675 -0.01408952633734 -0.39069089272734
F  -0.68251704328220  1.86219221482370  0.39160307190110
O   0.51893570820880  0.19702702281058 -1.96532747486704
```

CAM-B3LYP/def2-TZVP S_1 minimum used in the full-AH test

```
6
CF3NO CAM-B3LYP/def2-TZVP S1 minimum; E = -467.543456745211 Eh
C  -0.34204805228557  0.55332125880326  0.08478534025659
F   0.08784233196280  0.10546479457803  1.24384592726691
N   0.69287865263425  0.31606052401395 -0.89673261085789
F  -1.46418233048432 -0.07641562418101 -0.24844397316039
F  -0.61476699203946  1.85068948916487  0.18079456300040
O   0.60451039021231  0.61915155762090 -2.06307324650561
```

ωB97X-D4/def2-TZVP S_0 minimum

```
6
CF3NO omegaB97X-D4/def2-TZVP S0 minimum; E = -467.776953497820 Eh
C  -0.35900512131145  0.62055074928972  0.07110014499077
F   0.11985653192776 -0.01060214338946  1.13680586220308
N   0.78652513901191  0.71165715452615 -0.93986156098396
F  -1.41950538550977 -0.01644137700859 -0.39016653019934
F  -0.68462858067316  1.86693314983337  0.39364115033711
O   0.52099040655470  0.19617446674881 -1.97034306634766
```

ωB97X-D4/def2-TZVP S_1 minimum used in the full-AH test

```
6
CF3NO omegaB97X-D4/def2-TZVP S1 minimum; E = -467.720468247215 Eh
C  -0.34297923278551  0.55343942858780  0.08631403295269
F   0.08662399920887  0.10450249663241  1.25042441079942
N   0.70274896936491  0.31224126928736 -0.89826401639090
F  -1.46622402484552 -0.07946092646679 -0.25211744900937
F  -0.61351851722281  1.85535996810871  0.17920131194870
O   0.59758280628007  0.62218976385051 -2.06438229030053
```

Data-use statement

The 66 digitized band maxima are adapted from Mason[2] with permission from the Royal Society of Chemistry. Interpolated curves are guides between measured points and are not additional observations. Shifted and broadened calculated spectra are post-processing sensitivity tests and should not be presented as unshifted predictions.
